# Elucidating the genetics of grain yield and stress-resilience in bread wheat using a large-scale genome-wide association mapping study with 55,568 lines

**DOI:** 10.1038/s41598-021-84308-4

**Published:** 2021-03-04

**Authors:** Philomin Juliana, Ravi Prakash Singh, Jesse Poland, Sandesh Shrestha, Julio Huerta-Espino, Velu Govindan, Suchismita Mondal, Leonardo Abdiel Crespo-Herrera, Uttam Kumar, Arun Kumar Joshi, Thomas Payne, Pradeep Kumar Bhati, Vipin Tomar, Franjel Consolacion, Jaime Amador Campos Serna

**Affiliations:** 1 International Maize and Wheat Improvement Center (CIMMYT), Texcoco, Mexico; 2 Department of Plant Pathology, Wheat Genetics Resource Center, Kansas State University, Manhattan, KS USA; 3 Campo Experimental Valle de Mexico, Instituto Nacional de Investigaciones Forestales, Agricolas Y Pecuarias (INIFAP), Chapingo, Mexico; 4 CIMMYT, NASC Complex, New Delhi, India; 5 Borlaug Institute for South Asia (BISA), New Delhi, India; 6 Institute of Advanced Research, Gandhinagar, Gujarat India

**Keywords:** Agricultural genetics, Genetic association study, Genetic markers, Plant breeding, Quantitative trait

## Abstract

Wheat grain yield (GY) improvement using genomic tools is important for achieving yield breakthroughs. To dissect the genetic architecture of wheat GY potential and stress-resilience, we have designed this large-scale genome-wide association study using 100 datasets, comprising 105,000 GY observations from 55,568 wheat lines evaluated between 2003 and 2019 by the International Maize and Wheat Improvement Center and national partners. We report 801 GY-associated genotyping-by-sequencing markers significant in more than one dataset and the highest number of them were on chromosomes 2A, 6B, 6A, 5B, 1B and 7B. We then used the linkage disequilibrium (LD) between the consistently significant markers to designate 214 GY-associated LD-blocks and observed that 84.5% of the 58 GY-associated LD-blocks in severe-drought, 100% of the 48 GY-associated LD-blocks in early-heat and 85.9% of the 71 GY-associated LD-blocks in late-heat, overlapped with the GY-associated LD-blocks in the irrigated-bed planting environment, substantiating that simultaneous improvement for GY potential and stress-resilience is feasible. Furthermore, we generated the GY-associated marker profiles and analyzed the GY favorable allele frequencies for a large panel of 73,142 wheat lines, resulting in 44.5 million datapoints. Overall, the extensive resources presented in this study provide great opportunities to accelerate breeding for high-yielding and stress-resilient wheat varieties.

## Introduction

Boosting the grain yield (GY) potential and stress-resilience of bread wheat (*Triticum aestivum* L.) is critical for ensuring global food-security and meeting future demands^1–3^. Despite persistent breeding efforts, the low annual rate of GY increase (0.9%)^4^, GY stagnation patterns^5^, and the increasing threats of drought and heat stresses on wheat yields^6–9^, necessitate the complementation of conventional breeding approaches with genomic tools that can expedite the development of high-yielding and stress-resilient wheat varieties^10^. However, wheat GY has remained as an elusive trait for genomic-breeding owing to its quantitative genetic control involving many loci with small effects, a paucity of understanding about the genetic basis of GY, inconsistent GY quantitative trait loci (QTL) identified in different environments, low heritability of GY across environments, epistatic effects, genotype × environment interactions, etc.^11–14^.

Dissecting the genetic architecture of wheat GY, identifying molecular markers closely linked to GY QTL and understanding the genomic profiles of wheat lines for GY increasing/favorable alleles (FAs) are critical to accelerate the rate of genetic gain for GY, as they have the potential to improve parental choices and enhance the selection accuracy. While classic linkage-mapping studies have bolstered the identification of GY QTL^15–17^, they are limited by their ability to identify only the alleles differing between the parents and involve extended population development time^18^. In contrast, a genome-wide association study (GWAS) based on the linkage disequilibrium (LD) between the causal polymorphisms and markers has proven to be a powerful approach, as it eliminates the need for developing recombinant lines and utilizes historic recombination events in existing populations^19–21^.

To elucidate the genetic basis of GY and identify consistent GY-associated loci across multiple populations and environments, we have designed a comprehensive and large-scale GWAS using 105,000 GY observations on 55,568 wheat breeding lines that were evaluated in different sites, years, planting systems, irrigation systems and abiotic stresses by the International Maize and Wheat Improvement Center (CIMMYT) at its primary GY testing site, the Norman E. Borlaug Experimental Research Station, Ciudad Obregon, Mexico (27° 29′ N, 109° 56′ W) and national partners in eight countries including Afghanistan, India, Iran, Myanmar, Nepal, Pakistan, Portugal and Turkey. In addition to reporting hundreds of significant marker-GY associations, we have created a reference map with the GY-associated markers aligned to the bread wheat reference sequence (RefSeq v.1.0)^22^. Furthermore, we have generated the GY-associated marker profiles for 73,142 lines, which is currently the largest panel characterized for GY loci and we have also analyzed the FA frequencies for the GY-associated markers in the profiled lines.

## Results

### Grain yield datasets overview and statistical analysis

The GY datasets used in this study included the following:

1. *Stage 1 (S1) of yield testing- *This included datasets with 7,649–9,914 lines evaluated in the first stage/year of yield testing at Obregon, during the 2013–2014 (referred to as 1314) to 2018–2019 (referred to as 1819) crop cycles in the optimally irrigated-bed planting environment (S1 irrigated-BP). A combined dataset with the GY best-linear unbiased estimates (BLUEs) of all the 54,064 S1 lines across crop cycles (referred to as S1 irrigated-BP combined) was also used.
2. *Stage 2 (S2) of yield testing- *This included datasets with 960 to 1,092 lines evaluated in the second stage/year of yield testing, during the 2013–2014 to 2018–2019 crop cycles in six different management conditions at Obregon, namely the irrigated-BP (S2 irrigated-BP), irrigated-flat planting (S2 irrigated-FP), moderate-drought stress (S2 moderate-drought), severe-drought stress (S2 severe-drought), early-sown heat stress (S2 early-heat) and late-sown heat stress (S2 late-heat) environments. In addition, combined datasets with the GY BLUEs of all the S2 lines across crop cycles were also used and they included S2 irrigated-BP combined (6,229 lines), S2 irrigated-FP combined (6,220 lines), S2 moderate-drought combined (6,211 lines), S2 severe-drought combined (6,217 lines), S2 early-heat combined (5,151 lines) and S2 late-heat combined (4,084 lines) datasets.
3. *Stage 1 and 2 of yield testing- *This included the GY BLUEs of 960–1,092 lines evaluated in the irrigated-BP environment in both Stages 1 and 2 of yield testing at Obregon, during the 2012–2013 (referred to as 1213) to 2018–2019 crop cycles, resulting in the following datasets: S1 1213 and S2 1314 irrigated-BP, S1 1314 and S2 1415 irrigated-BP, S1 1415 and S2 1516 irrigated-BP, S1 1516 and S2 1617 irrigated-BP, S1 1617 and S2 1718 irrigated-BP, S1 1718 and S2 1819 irrigated-BP and a combined dataset of all the 6,229 lines evaluated in Stages 1 and 2 across crop cycles (referred to as S1 and S2 irrigated-BP combined).
4. *Stage 3 (S3) of yield testing- *This included 261–272 lines evaluated in the third stage/year of yield testing at Obregon, during the 2014–2015 (referred to as 1415) to 2017–2018 (referred to as 1718) crop cycles in the irrigated-BP (S3 irrigated-BP), severe-drought stress (S3 severe-drought) and late-sown heat stress (S3 late-heat) environments. In addition, combined datasets with the GY BLUEs of all the S3 lines across crop cycles were also used and they included S3 irrigated-BP combined (1,060 lines), S3 severe-drought combined (1,056 lines), and S3 late-heat combined (1,060 lines).
5. *South Asia Bread Wheat Genomic Prediction Yield Trials (SABWGPYTs)- *This included 471–512 lines evaluated during the 2014–2015 to 2018–2019 crop cycles in the irrigated-FP environments of Ludhiana, Jabalpur and Pusa in India. Combined datasets with the GY BLUEs of all the SABWGPYT lines were also used and they included 2,475 lines in Jabalpur combined, 2,469 lines in Ludhiana combined and 2,465 lines in Pusa combined.
6. *Elite Spring Wheat Yield Trials (ESWYTs)- *This included 33–50 lines evaluated during some of the crop cycles between 2003 and 2017 at 14 sites in eight countries including: Darul Aman, Puza-I-Esan and Shesham Bagh in Afghanistan; Karnal, Pantnagar and Pune in India; Safiabad in Iran; Kyaukme in Myanmar; Bhairahwa in Nepal; Pirsabak, Sakrand and Tandojam in Pakistan; Alentejo in Portugal and Diyarbakir in Turkey, resulting in combined datasets with 182–583 lines in the different sites﻿.

Statistical analysis of the GY data (Table S1) indicated that the mean GY from the 2013–2014 to the 2018–2019 cycles had increased by 17.2% in the S1 irrigated-BP, 24.5% in the S2 irrigated-BP, 25.6% in the S2 irrigated-FP, 52.7% in the S2 moderate-drought, 85.8% in the S2 severe-drought and 4.8% in the S2 early-heat environments. Across the different cycles in the S2 environments, the mean GY was the highest in the S2 irrigated-BP (6.7 ± 0.8 t/ha), S2 irrigated-FP (6.6 ± 0.7 t/ha) and S2 early-heat (6.4 ± 0.3 t/ha) environments. Among the S3 environments, the S3 irrigated-BP environment had the highest mean GY (7.0 ± 0.9 t/ha), that had increased by 35.4% from the 2014–2015 to the 2017–2018 cycles. In the SABWGPYT sites, the highest mean GY (7.3 ± 0.7 t/ha) was observed in Jabalpur and GY had increased by 8.2%, 26.4% and 60.4% from the 2014–2015 to the 2018–2019 cycles in Jabalpur, Ludhiana and Pusa, respectively.

### Marker densities and population structure

**Figure 1:**
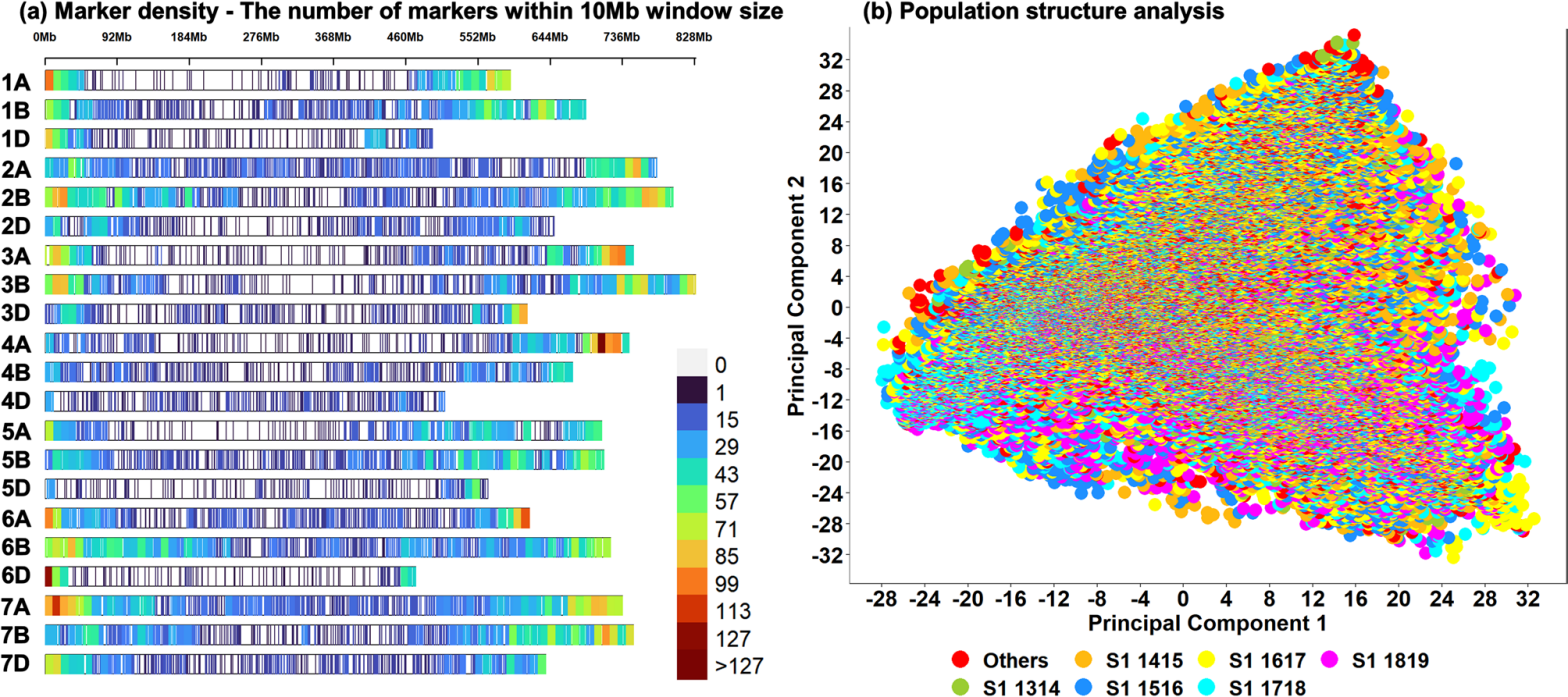
(**a**) Densities of 25,804 genotyping-by-sequencing markers in the reference bread wheat genome (RefSeq v1.0). The color key with marker densities indicates the number of markers within a window size of 10 Mb. (**b**) Population structure analysis of 55,568 lines. The plot of the first two principal components explaining 5.8% and 5.2% of the variation, respectively indicated weak population structure with high relatedness between the lines in the Stage 1 (S1) panels across crop cycles 2013–2014 (referred to as S1 1314) to 2018–2019 (referred to as S1 1819).

Marker densities of all the 25,804 genotyping-by-sequencing (GBS) markers used in this study, that were aligned to the RefSeq v1.0^22^, indicated that the densities at the telomeric and sub-telomeric regions were higher than the densities at the centromeric regions in all the chromosomes (Fig. 1a). The highest number of markers were present in the B-genome (45.2%), followed by the A-genome (36.5%) and the D-genome (16.9%). Population structure analysis of all the 55,568 lines in this study, using the first two principal components indicated moderate population structure, high diversity and relatedness between the lines in the S1 yield trials across years (Fig. 1b).

### Genome-wide association mapping for grain yield

**Figure 2:**
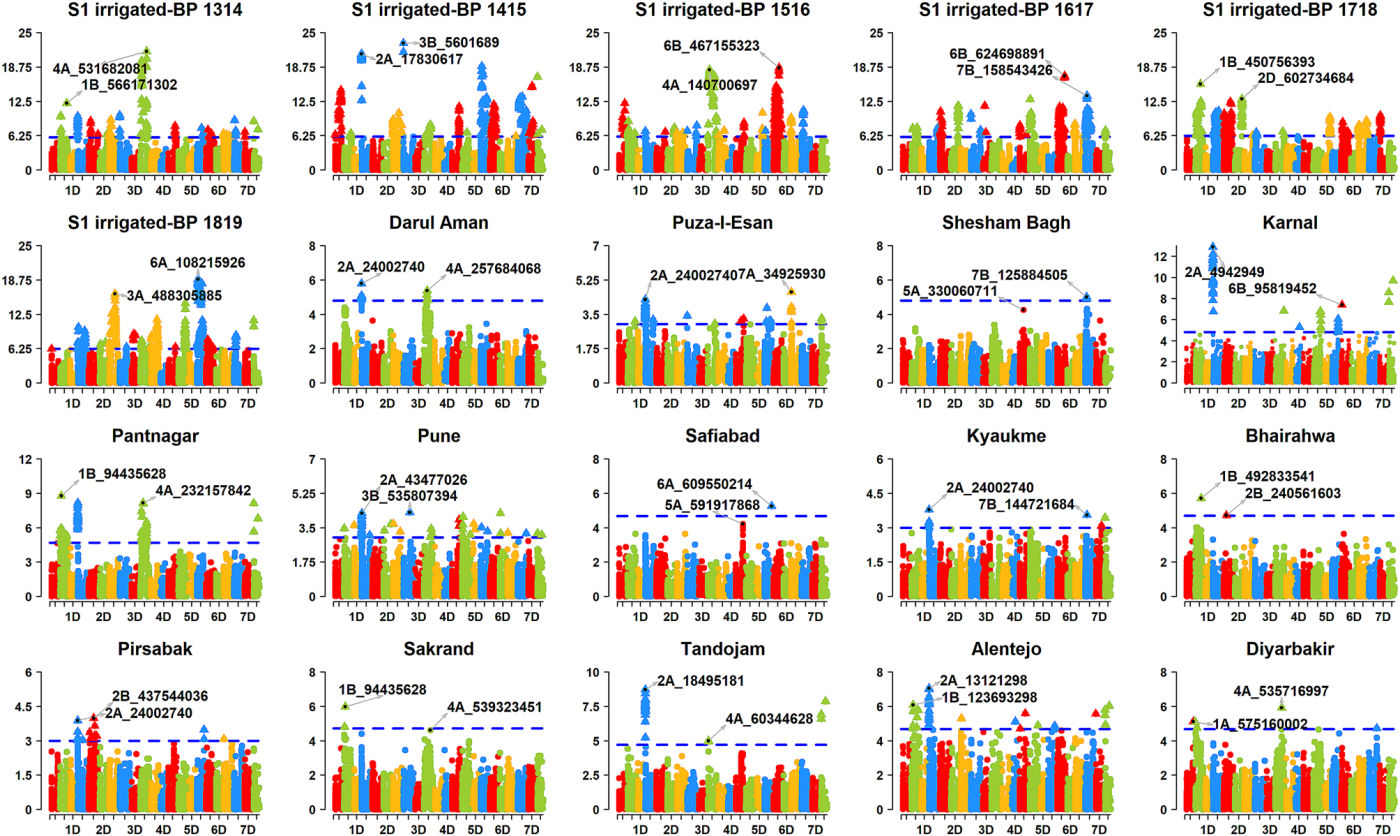
Markers significantly associated with grain yield in the Stage 1 (S1) yield trials evaluated in the irrigated-bed planting (BP) environment at Obregon (Mexico), during the 2013–2014 (S1 irrigated-BP 1314), 2014–2015 (S1 irrigated-BP 1415), 2015–2016 (S1 irrigated-BP 1516), 2016–2017 (S1 irrigated-BP 1617), 2017–2018 (S1 irrigated-BP 1718) and 2018–2019 (S1 irrigated-BP 1819) crop cycles and in the combined analysis of grain yield evaluated in the international sites (Darul Aman, Puza-I-Esan and Shesham Bagh in Afghanistan; Karnal, Pantnagar and Pune in India; Safiabad in Iran; Kyaukme in Myanmar; Bhairahwa in Nepal; Pirsabak, Sakrand and Tandojam in Pakistan; Alentejo in Portugal and Diyarbakir in Turkey) during some of the crop cycles between 2003 and 2017﻿. The chromosomes are shown in the x-axis and the − log_10_ *p* values in the y-axis. The threshold lines for the S1 yield trial datasets and the international sites datasets correspond to the threshold using the Bonferroni correction for multiple testing at an α level of 0.01 and 0.20, respectively.

**Figure 3:**
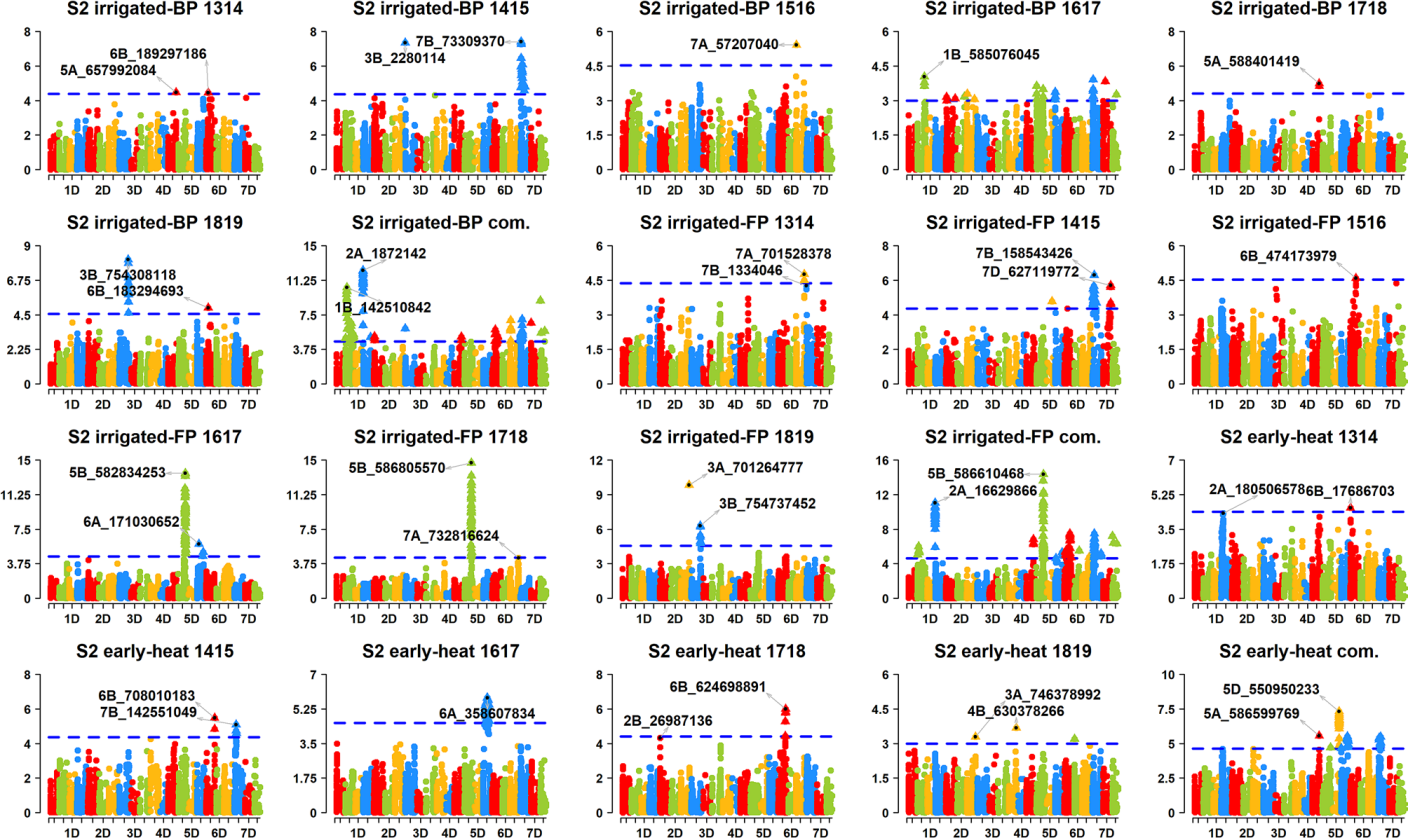
Markers significantly associated with grain yield in the Stage 2 (S2) yield trials evaluated in the irrigated-bed planting (S2 irrigated-BP), irrigated-flat planting (S2 irrigated-FP) and early-heat (S2 early-heat) environments at Obregon (Mexico), during the 2013–2014 (1314), 2014–2015 (1415), 2015–2016 (1516), 2016–2017 (1617), 2017–2018 (1718) and 2018–2019 (1819) crop cycles and in the combined (com.) analysis across crop cycles. The chromosomes are shown in the x-axis and the –log_10_ *p* values in the y-axis. The threshold lines correspond to the threshold using the Bonferroni correction for multiple testing at an α level of 0.20.

**Figure 4:**
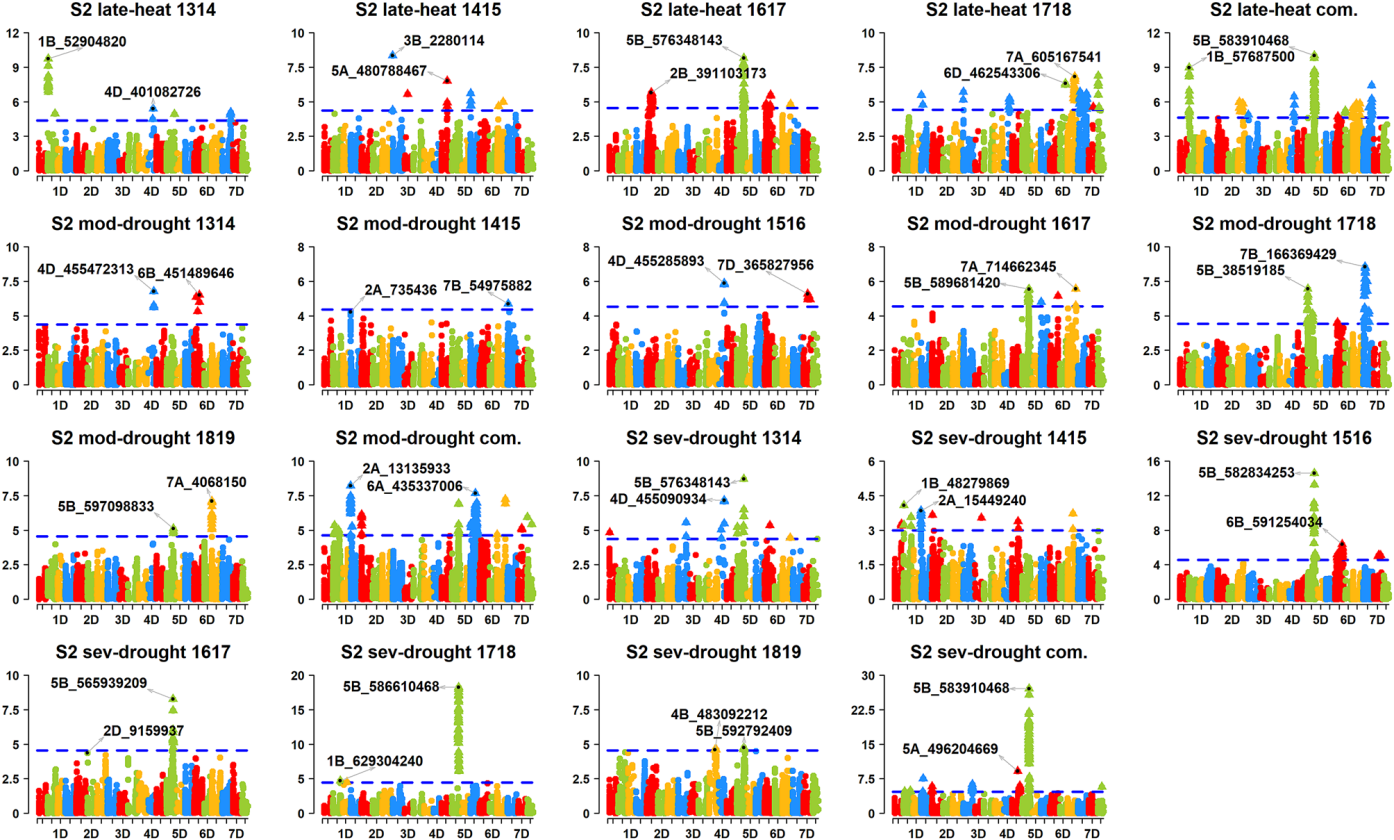
Markers significantly associated with grain yield in the Stage 2 (S2) yield trials evaluated in the late-heat (S2 late-heat), moderate-drought (S2 mod-drought) and severe-drought (S2 sev-drought) environments at Obregon (Mexico), during the 2013–2014 (1314), 2014–2015 (1415), 2015–2016 (1516), 2016–2017 (1617), 2017–2018 (1718) and 2018–2019 (1819) crop cycles and in the combined (com.) analysis across crop cycles. The chromosomes are shown in the x-axis and the − log_10_ *p* values in the y-axis. The threshold lines correspond to the threshold using the Bonferroni correction for multiple testing at an α level of 0.20.

**Figure 5:**
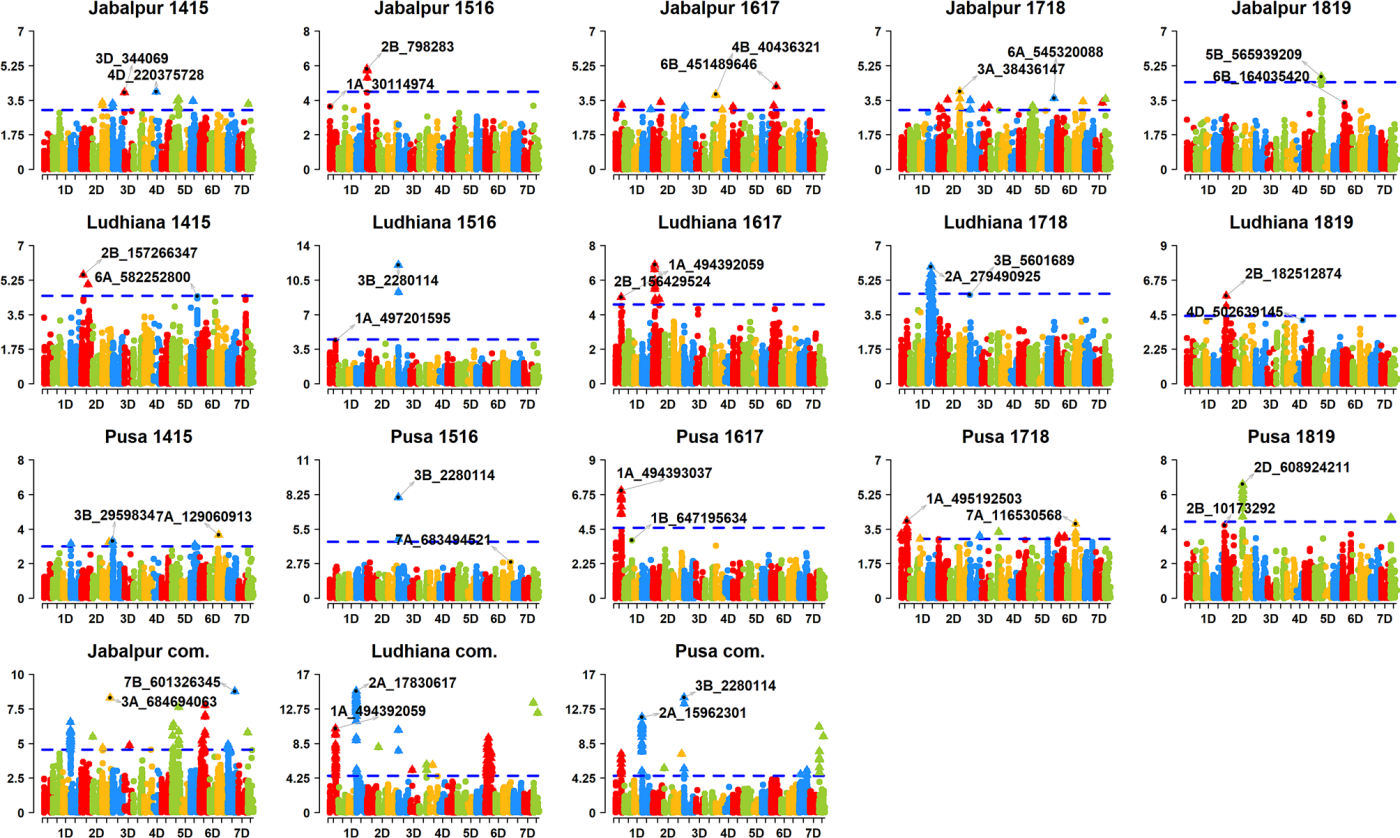
Markers significantly associated with grain yield evaluated in the irrigated-flat planting environments of Jabalpur, Ludhiana and Pusa (India), during the 2014–2015 (1415), 2015–2016 (1516), 2016–2017 (1617), 2017–2018 (1718) and 2018–2019 (1819) crop cycles and in the combined (com.) analysis across crop cycles. The chromosomes are shown in the x-axis and the − log_10_ *p* values in the y-axis. The threshold lines correspond to the threshold using the Bonferroni correction for multiple testing at an α level of 0.20.

**Figure 6:**
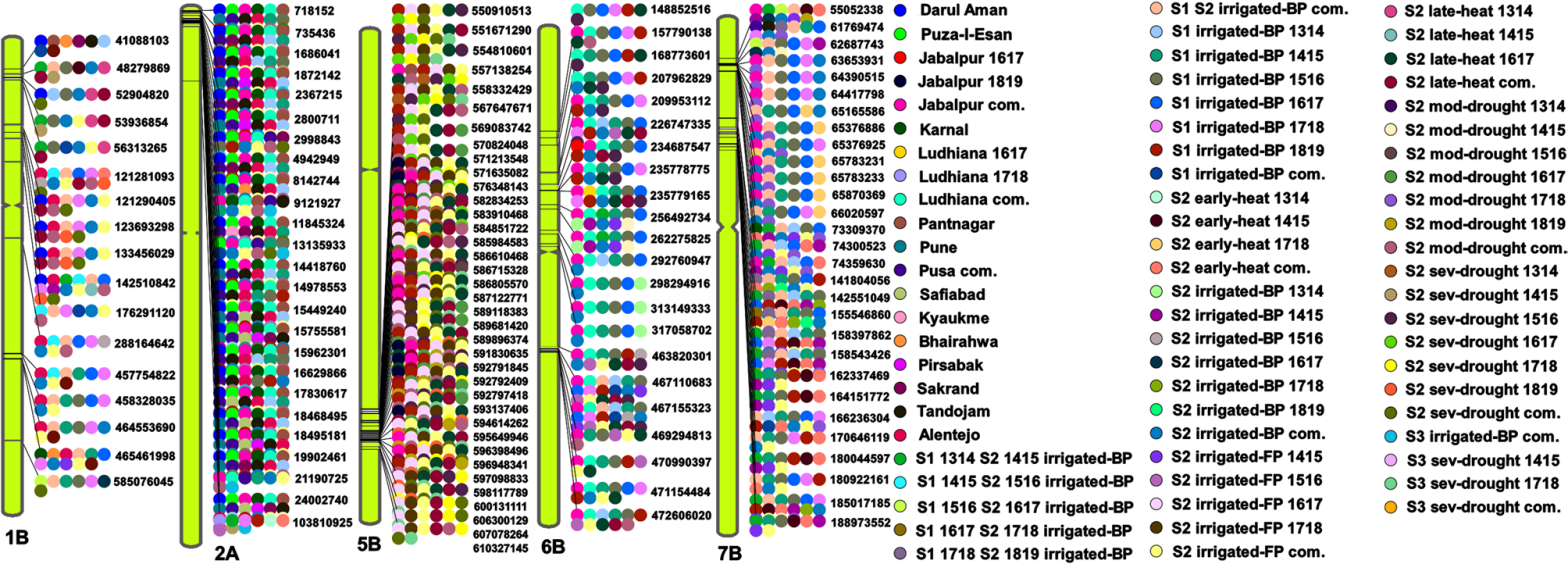
A reference grain yield-associated markers map aligned to the reference sequence of bread wheat (RefSeq v.1.0), with 130 grain yield-associated markers on chromosomes 1B, 2A, 5B, 6B and 7B, that were consistently associated in seven to twenty-four datasets. The reference map was visualized using Phenogram (http://visualization.ritchielab.org/phenograms/plot). The datasets in which the markers were significantly associated with grain yield included: Darul Aman (Afghanistan); Puza-I-Esan (Afghanistan); Jabalpur (India); Karnal (India); Ludhiana (India); Pantnagar (India); Pune (India); Pusa (India); Safiabad (Iran); Kyaukme (Myanmar); Bhairahwa (Nepal); Pirsabak (Pakistan); Sakrand (Pakistan); Tandojam (Pakistan); Alentejo (Portugal); best-linear unbiased estimates of Stage 1 (S1) and Stage 2 (S2) yield trials evaluated in the irrigated-bed planting (irrigated-BP) environment at Obregon (Mexico); Stage 1 (S1) yield trials evaluated in the irrigated-BP environment at Obregon; Stage 2 (S2) yield trials evaluated in the early-heat (S2 early-heat), irrigated-bed planting (S2 irrigated-BP), irrigated-flat planting (S2 irrigated-FP), late-heat (S2 late-heat), moderate-drought (S2 mod-drought) and severe-drought (S2 sev-drought) environments at Obregon; and Stage 3 (S3) yield trials evaluated in the irrigated-BP (S3 irrigated-BP) and severe-drought (S3 sev-drought) environments at Obregon. The names of the environments in the datasets are followed by the crop cycles: 2013–2014 (1314), 2014–2015 (1415), 2015–2016 (1516), 2016–2017 (1617), 2017–2018 (1718) and 2018–2019 (1819) and the combined (com.) analysis indicates the analysis across crop cycles.

We performed GWAS using GY data from 100 datasets and the filtered GBS markers (Table S2). We report the marker p-values from 734,304 tests of significance of associations between the markers and GY observations (Table S3). After Bonferroni correction for multiple testing, we obtained 1,413 markers that were significantly associated with GY in the different datasets (Figs. 2, 3, 4, 5, Fig. S1). This included 801 markers on all chromosomes that were significant in more than one dataset and the highest number of these markers were on chromosomes 2A (130), 6B (89), 6A (84), 5B (78), 1B (60) and 7B (56). For these consistent markers, we also report the genetic positions in the POPSEQ map^23^ (Table S4). In addition, we created a reference map aligned to the RefSeq v.1.0, with 159 GY-associated markers that were significant in seven to twenty-four datasets and observed that the highest number of those consistent markers were on chromosomes 5B (37), 7B (29) and 2A (25) (Fig. 6, Fig. S2). Furthermore, we used the LD between markers to designate 214 GY-associated LD-blocks (where each block constitutes markers with a pairwise R^2^ values greater than 0.9 and the p-value for the existence of LD equal to zero) and observed that 89.3% of the consistent LD-blocks in the irrigated-FP environment, 86.9% in the moderate-drought environment, 84.5% in the severe-drought environment, 100% in the early-heat environment and 85.9% in the late-heat environment overlapped with the consistent GY-associated LD-blocks identified in the irrigated-BP environment (Fig. S3). The key GY-associated LD-blocks on different homeologous chromosomes are highlighted below, along with their positions relative to the reported GY-associated and phenology-associated loci. The maximum additive effect (MAE) of a marker in the GY-associated LD-block within environments (not considering the combined datasets) is indicated in parenthesis after the dataset where the MAE was observed.

### Grain yield associations in group 1 homeologous chromosomes

On chromosome 1AL, *Yld.cim-1AL.1* to *Yld.cim-1AL.10* between 275,765,978 and 480,603,719 bps (78.6–83 cM) were associated with GY in the S1 irrigated-BP 1415 and S1 irrigated-BP 1516 (MAE of 166 kg/ha) datasets. In addition, *Yld.cim-1AL.11* between 493,823,499 and 495,283,626 bps (85.7 cM) was associated with GY in Ludhiana 1516, Ludhiana 1617, Ludhiana 1718, Ludhiana combined, Pusa 1617 (MAE of 188 kg/ha), Pusa 1718, Pusa combined, and S1 irrigated-BP combined datasets. Among these, *Yld.cim-1AL.4* and *Yld.cim-1AL.5* flanked marker *AX_110387060* associated with GY^24^, *Yld.cim-1AL.5* was close to marker *IWB42357* associated with spikes per square meter^17^ and *Yld.cim-1AL.2* to *Yld.cim-1AL.9* flanked the region between markers *Xgwm164* and *Xgwm135* that were associated with thousand kernel weight (TKW)^25^. *Yld.cim-1AL.11* was in the same position as *Qcim.1A.3* associated with GY in Ludhiana and Pusa^10^.

On chromosome 1BS, *Yld.cim-1BS.1* between 17,892,184 and 41,088,103 bps (37.4–44.4 cM) was associated with GY in the S1 irrigated-BP 1314 (MAE of 107.6 kg/ha), S1 irrigated-BP combined, Darul Aman, Diyarbakir, Pantnagar and Sakrand datasets. *Yld.cim-1BS.2* to *Yld.cim-1BS.6* and *Yld.cim-1BS.8* between 44,616,777 and 71,297,383 bps (44.4–63 cM) were all associated with GY in the S1 irrigated-BP 1314, S1 irrigated-BP 1516 and S2 late-heat 1314 (MAE of 148 kg/ha) datasets, while some of them were also associated with GY in the S2 late-heat combined, S2 irrigated-BP combined and S2 severe-drought combined datasets. *Yld.cim-1BS.10* between 121,281,093 and 142,510,842 bps (64 cM) was associated with GY in the S1 irrigated-BP 1718, S1 and S2 irrigated-BP combined, S2 irrigated-BP combined, S2 irrigated-FP combined, S2 severe-drought 1819 (MAE of 65 kg/ha), S2 moderate-drought combined, S2 severe-drought combined, S2 late-heat combined, Darul Aman and Alentejo datasets.

On chromosome 1BL, *Yld.cim-1BL.1* to *Yld.cim-1BL.3* between 288,164,642 and 533,399,880 bps (64–76.7 cM) were associated with GY in the S1 and S2 irrigated-BP combined and S1 irrigated-BP 1617 datasets, while some of them were also associated with GY in the S1 irrigated-BP 1718 (MAE of 74.2 kg/ha), S2 irrigated-BP combined, S2 irrigated-FP combined, S2 moderate-drought combined and Alentejo datasets. While *Yld.cim-1BL.1* was close to *QYld.agt-1B* reported to have a stable association with GY^16^, *Yld.cim-1BL.2* was in the same position as GY QTL *Qcim.1B.5*^10^.

### Grain yield associations in group 2 homeologous chromosomes

On chromosome 2AS, *Yld.cim-2AS.1* (0–8.9 cM) between 718,152 and 24,002,740 bps was significantly associated with GY in 29 datasets including S1 and S2 irrigated-BP combined, S1 irrigated-BP 1314, S1 irrigated-BP 1415, S1 irrigated-BP 1516 and S1 irrigated-BP 1819 (MAE of 110 kg/ha), S1 irrigated-BP combined, S2 irrigated-BP combined, S2 irrigated-FP 1516, S2 irrigated-FP combined, S2 moderate-drought 1415, S2 moderate-drought combined, S2 severe-drought 1415, S2 severe-drought combined, S3 irrigated-BP combined, Alentejo, Bhairahwa, Darul Aman, Jabalpur combined, Karnal, Kyaukme, Ludhiana combined, Pantnagar, Pirsabak, Pune, Pusa combined, Puza-I-Esan, Safiabad, Sakrand and Tandojam. *Yld.cim-2AS.1* was in the same position as *Qcim.2A.1* reported to be associated with GY in multiple environments^10^. On chromosome 2AL, *Yld.cim-2AL.1* and *Yld.cim-2AL.2* (68 cM) between 345,975,712 and 478,911,698 bps were associated with GY in the S1 irrigated-BP 1718 and Ludhiana 1718 (MAE of 144.5 kg/ha) datasets and flanked the cell wall invertase gene *TaCwi-A1* associated with kernel weight^26^.

On chromosome 2BS, *Yld.cim-2BS.2* to *Yld.cim-2BS.4* between 53,295,873 and 59,220,243 bps (60.5–61.4 cM) were associated with GY in the S1 1516 and S2 1617 irrigated-BP, S1 1718 and S2 1819 irrigated-BP, S1 and S2 irrigated-BP combined, S1 irrigated-BP 1617, S1 irrigated-BP 1718 (MAE of 64.3 kg/ha), S1 irrigated-BP combined and S2 irrigated-BP combined datasets. *Yld.cim-2BS.5* at 77.7 cM between 155,142,247 and 157,266,347 bps was associated with GY in the Ludhiana 1415, Ludhiana 1617 (MAE of 261 kg/ha) and Ludhiana 1819 datasets. On the centromeric region of chromosome 2B, *Yld.cim-2BS.7* and *Yld.cim-2BL.1* (78.5–84 cM) between 184,319,808 and 437,544,036 bps were associated with GY in the S2 late-heat 1617 (MAE of 98 kg/ha) and S2 late-heat combined datasets. Among these, *Yld.cim-2BS.2* to *Yld.cim-2BS.4* were in the same location as the *Photoperiod-B1 gene* associated with flowering time^27^, while *Yld.cim-2BS.5* to *Yld.cim-2BS.7* and *Yld.cim-2BL.1* flanked a region associated with TKW^28^. On chromosome 2DS, *Yld.cim-2DS.1* (13.8 cM) at 14,747,094 bps was associated with GY in the Jabalpur combined, Ludhiana combined and Pusa combined datasets and was in the same position as *Qcim.2D.1* associated with GY in Ludhiana and Pusa^10^. On chromosome 2DL, *Yld.cim-2DL.1* to *Yld.cim-2DL.7* (82.4–101 cM) between 572,256,847 and 620,582,835 bps were associated with GY in the S1 irrigated-BP 1617 and S1 irrigated-BP 1718 (MAE of 68.4 kg/ha) datasets, while some of them were also associated with GY in the S1 irrigated-BP 1516 dataset.

### Grain yield associations in group 3 homeologous chromosomes

On chromosome 3AS, *Yld.cim-3AS.1* and *Yld.cim-3AS.2* (3.6 cM) between 7,508,262 and 9,599,190 bps were associated with GY in the S1 irrigated-BP 1314, S1 irrigated-BP 1415 (MAE of 49.4 kg/ha) and S1 irrigated-BP 1516 datasets. In the centromeric region of chromosome 3A, *Yld.cim-3AS.4*, *Yld.cim-3AL.1* and *Yld.cim-3AL.2* (60.6 cM) between 142,889,830 and 434,822,203 bps were associated with GY in the S1 irrigated-BP 1314, S1 irrigated-BP 1516, S1 irrigated-BP 1819, S1 1213 and S2 1314 irrigated-BP, S1 and S2 irrigated-BP combined, S2 late-heat 1718 (MAE of 95 kg/ha) and S2 late-heat combined datasets. In addition, *Yld.cim-3AL.3* to *Yld.cim-3AL.7* (60.6–64.1 cM) between 435,447,288 and 525,879,700 bps were associated with GY in the S1 irrigated-BP 1415, S1 irrigated-BP 1516, S1 irrigated-BP 1819 (MAE of 76.7 kg/ha) and S1 and S2 irrigated-BP combined datasets. We observed that *Yld.cim-3AS.1* and *Yld.cim-3AS.2* flanked *Qcim.3A.1* associated with GY^10^ and a GY QTL linked to marker *Xbarc310*^29^, while *Yld.cim-3AS.4* flanked the *TaGS5-3A* gene associated with grain size^30^ and GY QTL linked to markers *Xbarc356*^31^, *Xbarc86*^32^ and *BE425222*^29^.

On chromosome 3BS, *Yld.cim-3BS.1* (0 cM) at 2,280,114 bps and *Yld.cim-3BS.2* (6.8 cM) at 5,601,689 bps were associated with GY in 14 datasets each, with nine common datasets including the S1 irrigated-BP 1314, S1 irrigated-BP 1415, S1 irrigated-BP 1617, S1 and S2 irrigated-BP combined, S2 irrigated-BP combined, Ludhiana 1516 (MAE of 302 kg/ha), Ludhiana combined, Pusa 1516 and Pusa combined datasets. On chromosome 3BL, *Yld.cim-3BL.1* (98.1 cM) between 754,308,118 and 754,737,452 bps, *Yld.cim-3BL.2* (98.1–102.2 cM) between 757,186,251 and 762,772,544 bps and *Yld.cim-3BL.3* (98.1 cM) between 757,480,752 and 757,480,826 bps were associated with GY in the S1 irrigated-BP 1516, S1 1718 and S2 1819 irrigated-BP, S2 irrigated-BP 1819, S2 irrigated-FP 1819 and S2 early-heat 1617 (MAE of 320 kg/ha) datasets. Among these, *Yld.cim-3BS.1* and *Yld.cim-3BS.2* were in the same position as GY QTL *Qcim.3B.1* and *Qcim.3B.2*, respectively^10^, while *Yld.cim-3BL.2* was in the same position as *Qcim.3B.6* associated with GY under early-heat^10^.

### Grain yield associations in group 4 homeologous chromosomes

On chromosome 4AS, *Yld.cim-4AS.1* to *Yld.cim-4AS.6* (23.2–28.2 cM) between 68,560,061 and 232,157,842 bps were all associated with GY in the S1 irrigated-BP 1516 dataset, while some of them were also associated with GY in the S1 irrigated-BP 1314 (MAE of 164 kg/ha), S1 irrigated-BP 1415, Darul Aman and Pantnagar datasets. On chromosome 4AL, *Yld.cim-4AL.1* and *Yld.cim-4AL.2* (29 cM) between 478,401,963 and 537,445,931 bps were associated with GY in the S1 irrigated-BP 1314 (MAE of 178 kg/ha), S1 irrigated-BP 1415, S1 irrigated-BP 1516, and Pantnagar datasets. Among these, *Yld.cim-4AL.1* and *Yld.cim-4AL.2* were in the region flanked by a TKW QTL, *QTgw.nfcri-4A*^33^ and *Yld.cim-4AL.2* was 70 Mb away from the GY-associated *TaCWI-4A* gene^34^.

On chromosome 4BS, *Yld.cim-4BS.1* to *Yld.cim-4BS.3* (59.7–65.7 cM) between 37,529,724 and 70,575,958 bps were significantly associated with GY in the S1 irrigated-BP 1516 (MAE of 146 kg/ha) and S1 irrigated-BP 1819 datasets. On chromosome 4DL, *Yld.cim-4DL.1* to *Yld.cim-4DL.3* (8–15.7 cM) between 386,212,705 and 455,618,800 bps were all associated with GY in the S2 late-heat 1314 and S2 late-heat 1718 (MAE of 103 kg/ha) datasets, while some of them were also associated with GY in the S2 moderate-drought 1314, S2 moderate-drought 1516 and S2 severe-drought 1314 datasets.

### Grain yield associations in group 5 homeologous chromosomes

On chromosome 5AL, *Yld.cim-5AL.1* to *Yld.cim-5AL.4* at 58.3 cM between 586,599,769 and 597,750,832 bps were all associated with GY in the S1 irrigated-BP 1415 (MAE of 62.7 kg/ha), S1 irrigated-BP 1516 and S2 irrigated-BP combined datasets, while some of them were also associated with GY in the S2 early-heat combined and S2 irrigated-FP combined datasets, and they flanked the vernalization gene *Vrn-A1*^35^. On chromosome 5BS, *Yld.cim-5BS.1* and *Yld.cim-5BS.2* (33.2–44.3 cM) between 28,753,553 and 38,519,185 bps were associated with GY in the S1 irrigated-BP 1617 and S2 moderate-drought 1718 (MAE of 74 kg/ha) datasets. *Yld.cim-5BS.3* and *Yld.cim-5BS.4* (46.8 cM) between 78,260,518 and 133,868,879 bps were associated with GY in the S1 irrigated-BP 1617 (MAE of 72.6 kg/ha) and Jabalpur combined datasets.

On chromosome 5BL, *Yld.cim-5BL.2* to *Yld.cim-5BL.21* (102.2–124.5 cM) between 550,910,513 and 610,327,145 bps were all associated with GY in the S2 severe-drought combined, S2 irrigated-FP 1617 and S2 irrigated-FP combined datasets, while some of them were associated with GY in the S2 severe-drought 1516, S2 severe-drought 1617, S2 severe-drought 1718 (MAE of 222 kg/ha), S2 severe-drought 1819, S3 severe-drought 1718, S2 moderate-drought 1617, S2 moderate-drought combined, S2 late-heat 1617, S2 late-heat combined, S1 irrigated-BP 1819, S2 irrigated-FP 1718 and Jabalpur combined datasets. Among these, *Yld.cim-5BS.1* to *Yld.cim-5BS.4* were in the position of the TKW associated *qTKW-5B.1*^25^; *Yld.cim-5BS.3* flanked a GY associated marker *wsnp_BQ166999B_Ta_2_1*^36^; *Yld.cim-5BL.6* and *Yld.cim-5BL.7* flanked the vernalization gene *Vrn-B1*^35^ and also *Qcim.5B.6.1* that was associated with GY under drought^10^ and *Yld.cim-5BL.21* was in the same position as *Qcim.5B.5.3* associated with GY^10^. On chromosome 5DL, *Yld.cim-5DL.1* to *Yld.cim-5DL.8* (222.4 cM) between 548,099,301 and 553,463,316 bps were associated with GY in the S1 irrigated-BP 1516, S1 irrigated-BP 1718 (MAE of 62.2 kg/ha) and S2 early-heat combined datasets.

### Grain yield associations in group 6 homeologous chromosomes

On chromosome 6A, *Yld.cim-6AS.2* to *Yld.cim-6AS.7* and *Yld.cim-6AL.1* to *Yld.cim-6AL.5* (53.8–64.4 cM) between 74,106,770 and 498,954,585 bps were all associated with GY in the S1 irrigated-BP 1415 and S1 irrigated-BP 1819 datasets, while several of them were also associated with GY in the S2 early-heat 1617 (MAE of 199 kg/ha) dataset. *Yld.cim-6AL.6* to *Yld.cim-6AL.8* (67.9 cM) between 520,667,476 and 540,151,122 bps were associated with GY in the S1 irrigated-BP 1314, S1 irrigated-BP 1415, S2 irrigated-BP 1314 (MAE of 83.9 kg), S2 early-heat combined, S1 1314 and S2 1415 irrigated-BP, Karnal and Pantnagar datasets. Among these *Yld.cim-6AS.8* flanked the *TaGW2-6A* gene associated with grain weight^37^; *Yld.cim-6AS.7* and *Yld.cim-6AL.4* flanked GY associated markers *RFL_Contig4632_1512* and *Kukri_c14877_303,* respectively^38^ and *Yld.cim-6AL.6* to *Yld.cim-6AL.8* were in the same position as GY QTL *Qcim.6A.7*^10^.

On the centromeric region of chromosome 6B, *Yld.cim-6BS.3* to *Yld.cim-6BS.19* and *Yld.cim-6BL.1* to *Yld.cim-6BL.8* (65––78 cM) between 135,263,307 and 515,290,507 bps were all associated with GY in the S1 irrigated-BP 1516 and Ludhiana combined datasets, while some of them were also associated with GY in the S1 irrigated-BP 1314, S1 irrigated-BP 1415, S1 irrigated-BP 1617, S1 irrigated-BP 1718, S1 irrigated-BP 1819, S1 irrigated-BP combined, S2 irrigated-BP 1314, S2 irrigated-BP combined, S2 irrigated-FP 1516, S2 irrigated-FP combined, S2 late-heat 1617, S2 late-heat combined, S2 moderate-drought combined, S2 severe-drought 1516, Jabalpur combined and Ludhiana 1617 (MAE of 134.2 kg/ha) datasets. While *Yld.cim-6BS.18* flanked the GY QTL *Qcim.6B.5.3*^10^; *Yld.cim-6BS.19* flanked the GY QTL *Qcim.6B.5.4*^10^ and the TKW associated *TaGW2-6B* gene^37^; *Yld.cim-6BS.8* and *Yld.cim-6BS.9* were in the same position as GY-associated marker *wPt8183*^39^ and *Yld.cim-6BL.1* flanked the GY associated *QYld.abrii-6B1.1*^40^.

On chromosome 6DL, *Yld.cim-6DL.1* (159.4 cM) between 460,481,447 and 460,856,226 bps was associated with GY in the S1 irrigated-BP 1819 and Ludhiana 1415 (MAE of 142 kg/ha) datasets, while *Yld.cim-6DL.2* (159.4 cM) between 462,303,873 and 462,543,306 bps was associated with GY in the S2 late-heat 1718 (MAE of 108.6 kg/ha) and in the S2 late-heat combined dataset. *Yld.cim-6DL.1* and *Yld.cim-6DL.2* flanked the TKW associated markers *AX_109481324*^24^ and *Ex_c7086_187*^41^.

### Grain yield associations in group 7 homeologous chromosomes

On chromosome 7AS, *Yld.cim-7AS.3* to *Yld.cim-7AS.6* (3.5–4.3 cM) between 3,634,691 and 4,463,061 bps were associated with GY in the S1 irrigated-BP 1516, S1 irrigated-BP 1617 and S1 irrigated-BP 1718 (MAE of 64 kg/ha) datasets. *Yld.cim-7AS.8* (54.7–68.5 cM) between 68,211,963 and 70,208,197 bps was associated with GY in the S1 irrigated-BP 1314 (MAE of 51.4 kg/ha), S1 irrigated-BP 1617, S1 irrigated-BP 1718 and S2 irrigated-BP combined datasets. *Yld.cim-7AS.9* (68.5 cM) between 75,305,239 and 75,820,143 bps was associated with GY in the S1 irrigated-BP 1415, S1 irrigated-BP 1718, S2 irrigated-BP combined, S2 early-heat combined, S2 irrigated-BP 1718 and S3 late-heat 1718 (MAE of 181 kg/ha) datasets. Among these, *Yld.cim-7AS.8* and *Yld.cim-7AS.9* flanked the vernalization gene *Vrn-A3*^42^, while *Yld.cim-7AS.1* to *Yld.cim-7AS.7* flanked a GY QTL *Qcim.7A.2*^10^ and *Yld.cim-7AS.3* was close to a TKW associated marker *BS00022146*^43^.

On chromosome 7BS, *Yld.cim-7BS.1 to Yld.cim-7BS.11* (66.5–76.4 cM) between 55,052,338 and 196,382,351 bps were associated with GY in several datasets, including the S1 irrigated-BP 1314, S1 irrigated-BP 1415, S1 irrigated-BP 1516, S1 irrigated-BP 1617, S1 irrigated-BP 1718, S1 irrigated-BP 1819, S1 1314 and S2 1415 irrigated-BP, S1 1718 and S2 1819 irrigated-BP, S1 and S2 irrigated-BP combined, S2 early-heat 1415, S2 early-heat combined, S2 irrigated-BP 1415, S2 irrigated-BP combined, S2 irrigated-FP 1415, S2 irrigated-FP combined, S2 moderate-drought 1718, S2 late-heat 1718 (MAE of 149 kg/ha) and Jabalpur combined datasets. Among these, *Yld.cim-7BS.1* to *Yld.cim-7BS.4* flanked a GY QTL *Qcim.7B.2*^10^, *Yld.cim-7BS.3* and *Yld.cim-7BS.4* flanked the sucrose synthase gene *TaSus1-7B* associated with TKW^44^ and *Yld.cim-7BS.6* was in the same position as *QGWt.ara-7B.1* associated with grain weight^45^.

On chromosome 7DS, *Yld.cim-7DS.3* and *Yld.cim-7DS.4* (61.4 cM) between 55,529,933 and 59,096,036 bps were associated with GY in the S1 irrigated-BP 1415, S1 irrigated-BP 1718 (MAE of 51 kg/ha), S1 and S2 irrigated-BP combined and S2 early-heat combined datasets and were in the position of the vernalization gene *Vrn-D3*^46^ and a QTL controlling grain weight^47^.

### Genomic profiling of lines for grain yield favorable alleles

**Figure 7:**
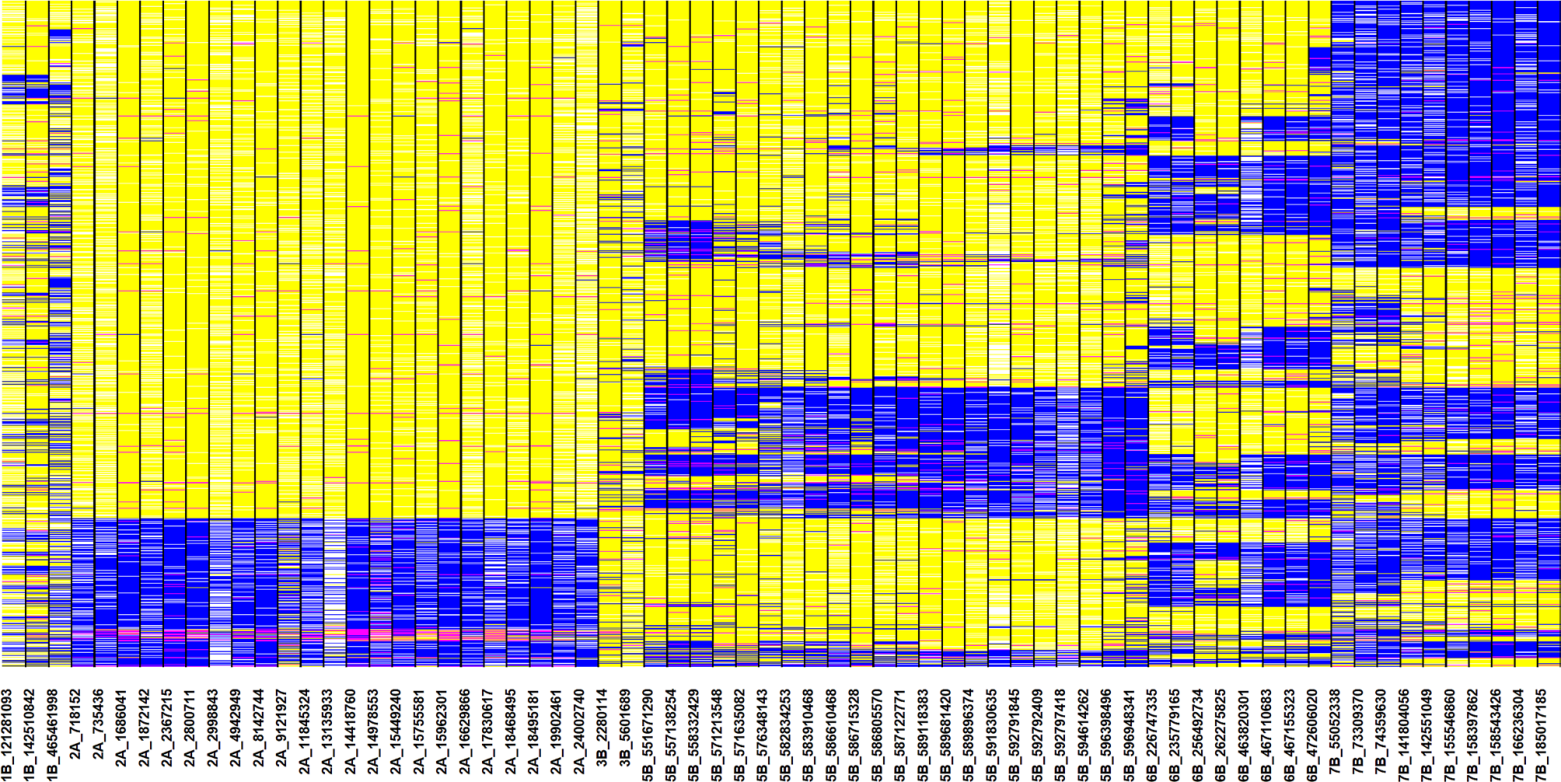
Genomic profiles of 73,142 wheat lines for 68 markers on chromosomes 1B, 2A, 3B, 5B, 6B and 7B that were associated with grain yield in 11–24 datasets. The yellow color indicates the favorable allele (allele that has an increasing effect on grain yield), the blue color indicates the non-favorable allele (allele that has a decreasing effect on grain yield), the magenta color indicates the heterozygote and the white color indicates missing data. For 56 of the 68 markers, we observed that greater than 50% of the lines had favorable alleles.

We profiled 73,142 wheat lines for the 801 consistent GY-associated markers, resulting in 44,554,376 datapoints (https://hdl.handle.net/11529/10548546). However, considering only the 16,401 and 38,743 lines that had greater than 90% and 80% non-missing data, respectively, 15,084 lines (92%) and 35,071 lines (90.5%) had FAs in greater than 50% of the GY-associated markers, respectively. The lines that had a high percentage of FAs (80.2–83%) and were also high yielding (GY between 8.2 and 8.6 t/ha) included PFAU/MILAN/3/BABAX/LR42//BABAX/11/CROC_1/AE.SQUARROSA(213)//PGO/10/ATTILA*2/9/KT/BAGE//FN/U/3/BZA/4/TRM/5/ALDAN/6/SERI/7/VEE#10/8/OPATA/12/2*KUTZ (GID8502028), KACHU/SAUAL//PRL/3/2*SUP152*2/TECUE #1 (GID8508887), MUCUY*2/COPIO (GID8506188), SUP152*2/TECUE#1//COPIO (GID8506937), BOKOTA/MUCUY (GID8239262), SUP152/4/WHEAR/KIRITATI/3/C80.1/3*BATAVIA//2*WBLL1*2/5/SWSR22T.B./2*BLOUK#1//WBLL1*2/KURUKU (GID8501303) and MELON//FILIN/MILAN/3/FILIN/4/TRCH/SRTU//KACHU*2/5/TAITA (GID8508936). We also examined the profiles of lines for the 68 markers that were significantly associated with GY in greater than 10 datasets and observed that for 56 of those markers, greater than 50% of the lines had FAs. However, some markers like 7B_74359630, 7B_73309370 and 7B_55052338 had FAs in only 18.4–24.6% of the lines (Fig. 7). Overall, we observed that 551 (68.8%) of the 801 markers had FAs in more than 50% of the lines. The highest number of markers with FAs less than 50% were on chromosomes 6A, 7B, 5B and 2D (Fig. S4–S10).

## Discussion

We have performed the largest GWAS for wheat GY using a massive panel of wheat breeding lines evaluated in multiple environments and have reported consistent LD-blocks associated with GY in the irrigated-BP (199 LD-blocks), irrigated-FP (56 LD-blocks), moderate-drought (61 LD-blocks), severe-drought (58 LD-blocks), early-heat (48 LD-blocks) and late-heat (71 LD-blocks) environments and target sites in different countries (97 LD-blocks). These include several novel loci and previously reported loci, that together provide important insights into the quantitative genetic architecture of GY. However, it should be noted that the GY-associated LD-blocks reported in this study are only based on pairwise R^2^ values greater than 0.9 and are not independant. Hence, several LD-blocks could be in LD with the same QTL, due to the high LD in wheat^10^. The association of *Yld.cim-1BS.10* with GY in all the environments mentioned above, indicates its exceptional value in breeding for GY in the optimum, drought and heat stressed environments. In addition, *Yld.cim-2AS.1* that was associated with GY in the optimum, drought-stressed and target environments and also associated with GY in the highest number of datasets in this study, was in the position of the 2NS translocation from *Aegilops ventricosa*. This is an invaluable region in wheat breeding, because of its additional favorable effect on resistance to rusts and wheat blast diseases, lodging tolerance, etc.^10^. We also observed that some LD-blocks were in the location of the vernalization genes and the *Photoperiod-B1* gene. These have to be considered cautiously as they might not be associated with GY per se, but are significant because of their effect on flowering time that determines GY and stress-adaptation^48^. While accounting for flowering time in GWAS is possible, the large populations used in this study comprised lines with a wide range in their flowering time and we were interested in exploiting this variation to understand the flowering time-linked genes that had an indirect effect on GY.

A remarkable observation in our study was that greater than 84% of the LD-blocks identified in the stressed-environments overlapped with the consistent LD-blocks identified in the irrigated-BP environment. This substantiates the positively correlated performance of lines in optimum and stressed environments, indicating that simultaneous improvement for GY potential and stress-resilience is feasible^49^. Considering the target sites in different countries, we observed that 93.8% of the consistent LD-blocks identified in them, overlapped with those identified in the irrigated-BP environment in CIMMYT’s key selection environment, Obregon, thereby providing compelling evidence to the shared genetic basis of GY in CIMMYT’s selection environment and target environments in different countries.

While it is interesting that only 56.7% of all the significant markers identified in this study were significant in more than one dataset, we also observed that the highest number of datasets where a marker was significantly associated with GY was only 24, thereby accentuating the persistent challenge of identifying consistent GY-associated markers in different environments. Furthermore, our observation of a large variation in the additive effect of markers in different environments (average range in the additive effect of a marker across environments was 59.1 kg/ha and the maximum range was 245 kg/ha) clearly indicated the inconsistency of marker effects, which complicates their deployment in marker-assisted selection and genomic selection across years^10,50–52^. We also observed that among the 2,389 significant marker-dataset associations within environments and cycles, the marker additive effects were between 300 and 320 kg/ha in 0.38% (9) of the marker-dataset associations, between 200 and 299 kg/ha in 1.97% (47) of the marker-dataset associations, between 100 and 199 kg/ha in 26.4% (630) of the marker-dataset associations and between 23 and 99 kg/ha in 71.3% (1,703) of the marker-dataset associations. This substantiates that while GY is pre-dominantly controlled by many small-effect loci, there are some moderate to large-effect loci, that can be utilized in selections.

We have also successfully utilized the Bayesian-information and Linkage-disequilibrium Iteratively Nested Keyway method^53^ for GWAS in the S1 irrigated-BP combined dataset with  greater than 50,000 lines. While it involved a significantly low computation time (~ 8 h), we also observed that 42.7% markers identified by this method were also identified in other datasets using the mixed linear model implemented in TASSEL (Trait Analysis by aSSociation Evolution and Linkage). We have also developed a reference map with consistent GY-associated markers aligned to the RefSeq v1.0, which is an exemplary resource to the wheat community for comparing GY-associated markers in different populations and environments. Finally, we have also reported the genomic profiles of GY-associated markers for the largest panel of wheat lines, which indicated that the CIMMYT wheat germplasm is rich in GY FAs and a trove for breeders to choose parents and design strategic crosses based on complementary GY alleles at desired loci. Overall, this study has extended the knowledge on the genetic architecture of wheat GY and the extensive resources presented provide significant opportunities to accelerate breeding for high-yielding and stress-resilient wheat varieties.

## Online methods

### Grain yield testing trials, environments, and designs

Grain yield (GY) was measured as the harvested grain weight on a plot-basis and the GY testing trials used in this study comprised the following:

1. *Stage 1 GY testing trials*
  These trials comprised greater than 7,000 lines in the first year of GY testing at Obregon (Mexico), selected each year from about 70,000 head-row derived lines that were developed using the selected-bulk breeding method. In this method, all the early-generations were selected for agronomic type, phenology, rust resistance, grain size and health, spike fertility and tillering capacity and spikes from the selected plants were bulked until the F4, F5, or F6 stage (depending on the cross and the breeding shuttle logistics), from which the head-rows were obtained and then selected again for the aforementioned traits. The Stage 1 GY testing trials used in this study were evaluated in an irrigated-bed planting (S1 irrigated-BP) environment, where the lines were planted during the optimum planting time (the last 8–10 days of November to the 1^st^ week of December) on raised beds in optimally irrigated environments that received a total of about 500 mm of water in five irrigations.
  The GY evaluation plot size was 4.8 m^2^ and the lines were sown in three rows over each of the two beds that were 80 cm wide. The lines were replicated twice in an alpha-lattice design, with each of the > 300 trials comprising 28 lines and two high-yielding check varieties arranged in six blocks. The lines were evaluated during the 2013–2014 (referred to as 1314) to the 2018–2019 (referred to as 1819) crop cycles as follows: (a) Stage 1 1314 comprising 7,649 lines evaluated during the 2013–2014 cycle (b) Stage 1 1415 comprising 9,089 lines evaluated during the 2014–2015 cycle (c) Stage 1 1516 comprising 9,501 lines evaluated during the 2015–2016 cycle (d) Stage 1 1617 comprising 9,914 lines evaluated during the 2016–2017 cycle (e) Stage 1 1718 comprising 8,984 lines evaluated during the 2017–2018 cycle (f) Stage 1 1819 comprising 8,927 lines evaluated during the 2018–2019 cycle.
2. *Stage 2 GY testing trials*
  These trials comprised 1,092 lines in the second year of GY testing at Obregon, that were selected from the lines in Stage 1 for high GY, good resistance to stem and stripe rust, acceptable agronomic type, phenology and end-use quality. The lines were sown in three replications in an alpha-lattice design and each of the 39 trials comprised 28 lines and two high-yielding check varieties arranged in six blocks, during the 2013–2014 to 2018–2019 crop cycles in six managed environments including:
  (i) *Stage 2 irrigated-bed planting (S2 irrigated-BP) environment*—In this environment, GY potential was evaluated for lines planted on raised beds during the optimum planting time (the third week of November) and they received an optimum irrigation of about 500 mm of water in total from five irrigations. The GY evaluation plot size was 4.8 m^2^ and the lines were sown in three rows over each of the two beds that were 80 cm wide.
  (ii) *Stage 2 irrigated-flat planting (S2 irrigated-FP) environment*—In this environment, GY potential was evaluated for lines planted on flat seed beds during the optimum planting time (the third week of November) and they received an optimum irrigation of about 500 mm of water in total from five or more irrigations. The GY evaluation plot size was 5.46 m^2^, and the lines were sown in six rows that were 18 cm apart and 4.2 m in length.
  (iii) *Stage 2 moderate-drought stress (S2 moderate-drought) environment*—In this environment, the GY under moderate-drought stress was evaluated for lines planted on raised beds during the optimum planting time (the third week of November) and they received a reduced irrigation of about 200 mm of water in total from two irrigations. The GY evaluation plot size, the number of rows and beds were similar to the S2 irrigated-BP environment.
  (iv) *Stage 2 severe-drought stress (S2 severe-drought) environment*—In this environment, the GY under severe-drought stress was evaluated for lines planted on flat seed beds during the optimum planting time (the third week of November) and they received water through drip irrigation to adjust the total available soil water to about 180 mm. The GY evaluation plot size was 5.85 m^2^, and the lines were sown in six rows that were 18 cm apart and 4.5 m in length.
  (v) *Stage 2 early-sown heat stress (S2 early-heat) environment*—In this environment, the GY under heat stress during the juvenile growth stage was evaluated for lines planted on raised beds about 30 days before the optimum planting time (around the 20th of October) and they received an optimum irrigation of about 500 mm of water in total from five irrigations. The Stage 2 panels were evaluated in the early-heat environment during the 2013–2014 to 2018–2019 crop cycles, except in the 2015–2016 cycle. The GY evaluation plot size, the number of rows and beds were similar to the S2 irrigated-BP environment.
  (vi) *Stage 2 late-sown heat stress (S2 late-heat) environment*—In this environment, the GY under heat stress during the heading and grain-filling stages was evaluated for lines planted on raised beds about 90 days after the optimum planting time (the last week of February) and they received an optimum irrigation of about 500 mm of water in total from five irrigations. The Stage 2 panels were evaluated in the late-heat environment during the 2013–2014 to 2017–2018 crop cycles, except the 2015–2016 cycle. The GY evaluation plot size, the number of rows and beds were similar to the S2 irrigated-BP environment.
  After removing the lines with missing data, we obtained 961 lines in the 2013–2014 cycle, 1,012 lines in the 2014–2015 cycle, 1,052 lines in the 2015–2016 cycle, 1,040 lines in the 2016–2017 cycle, 1,092 lines in the 2017–2018 cycle and 1,078 lines in the 2018–2019 cycle.
3. *Stage 3 GY testing panels*
  These trials comprised 280 lines in the third year of GY testing in Obregon that were selected from the lines in Stage 2 for high GY in different environments, good resistance to leaf rust, stem rust, stripe rust, Fusarium head blight, Septoria tritici blotch and spot blotch, acceptable agronomic type, phenology and end-use quality. The lines were also sown in three replications in an alpha-lattice design (10 trials, with each trial comprising 28 lines and two high-yielding check varieties in six blocks), during the 2014–2015 to 2017–2018 crop cycles in three managed environments similar to that in Stage 2, including the irrigated-bed planting (S3 irrigated-BP), severe-drought stress (S3 severe-drought) and late-sown heat stress (S3 late-heat) environments.
  After removing the lines with missing data, we obtained 261 lines in the 2014–2015 cycle, 263 lines in the 2015–2016 cycle, 272 lines in the 2016–2017 cycle and 264 lines in the 2017–2018 cycle.
4. *South Asia Bread Wheat Genomic Prediction Yield Trials (SABWGPYTs)*
  These trials comprised 540 lines in each cycle and were selected from the Stage 2 of yield testing in Obregon for most of the traits that were used to select lines from Stage 2 for Stage 3 of yield testing. The lines were evaluated for their GY potential in flat seed beds with two replications during the 2014–2015 to 2018–2019 crop cycles and the alpha-lattice trial design was used. Evaluations were done at the research stations of the Borlaug Institute for South Asia in the following main wheat growing regions of India: (1) Jabalpur, Madhya Pradesh (23° 10′ N, 79° 55′ E, representing the Central Zone) (2) Ludhiana, Punjab (30° 54′ N, 75° 51′ E, representing the North-Western Plain Zone) and (3) Pusa, Bihar (25° 59′ N, 85° 41′ E, representing the North-Eastern Plain Zone).
  After removing the lines with missing data, we obtained 504 lines in the 2014–2015 cycle, 487 lines in the 2015–2016 cycle, 512 lines in the 2016–2017 cycle, 501 lines in the 2017–2018 cycle and 471 lines in the 2018–2019 cycle.
5. *Elite Spring Wheat Yield Trial (ESWYTs)*
  These trials comprised 50 lines in each cycle, with 46 breeding lines, one local check and three CIMMYT checks. The lines were selected for high and stable yields relative to checks from the irrigated trials at Obregon and South Asia, in addition to good to moderate drought and heat tolerance. Evaluations were done in an alpha-lattice design with two replications during some cycles between 2003 and 2017 by national partners at 14 sites including: (1) Darul Aman, Afghanistan (34° 33′ N, 69° 12′ E) at the Darul Aman Research Station for 535 lines (2) Shesham Bagh, Afghanistan (34° 25′ N, 70° 27′ E) for 536 lines (3) Puza-I-Esan, Afghanistan (36° 5′ N, 68° 39′ E) at the Puza-I-Esan Agricultural Research Farm for 318 lines (4) Karnal, India (29° 40′ N, 77° 2′ E) at the Indian Institute for Wheat and Barley Research for 583 lines (5) Pantnagar, India (29° 0′ N, 79° 30′ E) at the G.B. Pant University of Agriculture and Technology for 447 lines (6) Pune, India (18° 4′ N, 74° 21′ E) at the Agharkar Research Institute for 532 lines (7) Safiabad, Iran (32° 16′ N, 48° 25′ E) at the Seed and Plant Improvement Institute for 492 lines (8) Kyaukme, Myanmar (23° 2′ N, 95° 28′ E) at the Agriculture Research Farm for 182 lines (9) Bhairahwa, Nepal (27° 30′ N, 83° 27′ E) at the National Wheat Research Program for 542 lines (10) Pirsabak, Pakistan (33° 59′ N, 71° 59′ E) for 579 lines (11) Sakrand, Pakistan (26° 3′ N, 68° 3′ E) for 353 lines (12) Tandojam, Pakistan (25° 23′ N, 68° 24′ E) for 453 lines (13) Alentejo, Portugal (38° 54′ N, 7° 9′ W) at the National Research Institute for 322 lines (14) Diyarbakir, Turkey (37° 55′ N, 40° 12′ E) at the International Agricultural Research and Training Center for 365 lines.

### Best linear unbiased estimates and statistical analysis of the grain yield data

The best linear unbiased estimates (BLUEs) for GY in each of the panels and environments for Stages 1, 2 and 3 of yield testing and the SABWGPYTs were calculated using the ASREML statistical package^54^, using the following mixed model:

1 $$y_{ijkl} = \mu + g_{i} + t_{j} + r_{k\left( j \right)} + b_{{l\left( {jk} \right)}} + \varepsilon_{ijkl}$$

where $y_{ijkl}$ was the observed GY, μ was the overall mean, $g_{i}$ was the fixed effect of the genotype, $t_{j}$ was the random effect of the trial that was independent and identically distributed (IID) ($t_{j} \sim N \left( {0, \sigma_{t}^{2} } \right)$), $r_{k\left( j \right)}$ was the random effect of the replicate within the trial that was IID $(r_{k\left( j \right)} \sim N \left( {0, \sigma_{r}^{2} } \right))$, $b_{{l\left( {jk} \right)}}$ was the random effect of the incomplete block within the trial and the replicate that was IID ($b_{{m\left( {jk} \right)}} \sim N \left( {0, \sigma_{b}^{2} } \right))$ and $\varepsilon_{ijkl}$ was the residual that was IID $(\varepsilon_{ijkl} \sim N \left( {0, \sigma_{\varepsilon }^{2} } \right))$.

We also obtained the GY BLUEs for the 1,092 lines that were evaluated in both Stages 1 and 2 using the random effect of the year in Eq. (1), resulting in GY BLUEs for the lines evaluated in the following cycles: S1 1213 and S2 1314, S1 1314 and S2 1415, S1 1415 and S2 1516, S1 1516 and S2 1617, S1 1617 and S2 1718, S1 1718 and S2 1819. In addition, for the combined datasets comprising all the lines in the Stage 1–3 trials, the SABWGPYTs and the ESWYTs, we obtained the GY BLUEs across years with the random effect of the year included in Eq. (1). The GY BLUEs within each panel and environment were used to calculate the maximum, mean, median, minimum, range, standard deviation, standard error of the mean and variance of GY in the different datasets. We also obtained the percentage increase in GY across years in the Stage 1–3 trials, SABWGPYTs and ESWYTs using the initial year as the base year.

### Genotyping

The genotyping-by-sequencing (GBS) method^55^ was used to genotype all the lines used in this study and the single nucleotide polymorphisms (SNP) were called using the TASSEL (Trait Analysis by aSSociation Evolution and Linkage) version 5 GBS pipeline^56^.The discovery of SNPs was done using a minor allele frequency of 0.01, resulting in 13,082,477 GBS tags that were aligned to RefSeq v1.0 using Bowtie2^57^ with an overall at an alignment rate of 68.98%. The SNPs were then filtered for Fisher’s exact test (*p* < 0.001), inbred coefficient (> 80%) and Chi Square (expected inbreeding of 96%), and we obtained 89,863 SNPs that passed at least one of these filters. These markers were then filtered in each individual panel and in the combined panels and markers with greater than 60% missing data, less than 5% minor allele frequency and greater than 10% heterozygosity were removed. Similarly, the lines with greater than 50% missing data were removed, and the total number of markers and lines used are given in Table S3. Marker imputation was done using Beagle version 4.1 with default settings, except for the ‘err’ parameter which was set to 0.1 and the ‘number of iterations’ which was set to 1,055^58^.

### Genome‑wide association mapping and reference map with grain yield associated markers

We performed GWAS for GY in the following 100 datasets: (1) seven S1 yield trial datasets including the S1 irrigated-BP 1314, S1 irrigated-BP 1415, S1 irrigated-BP 1516, S1 irrigated-BP 1617, S1 irrigated-BP 1718, S1 irrigated-BP 1819 datasets and the S1 irrigated-BP combined dataset, (2) thirty-nine S2 yield trial datasets including the S2 1314, S2 1415, S2 1516, S2 1617, S2 1718 and S2 1819 trials evaluated in six environments and the combined datasets, (3) seven S1 and S2 GY BLUEs datasets including the S1 1213 and S2 1314, S1 1314 and S2 1415, S1 1415 and S2 1516, S1 1516 and S2 1617, S1 1617 and S2 1718, S1 1718 and S2 1819 and S1 and S2 GY BLUEs combined dataset, (4) fifteen S3 yield trial datasets including the S3 1415, S3 1516, S3 1617 and S3 1718 trials evaluated in three environments and the combined datasets, (5) eighteen SABWGPYT datasets including SABWGPYT 1415, SABWGPYT 1516, SABWGPYT 1617, SABWGPYT 1718 and SABWGPYT 1819 and the combined datasets evaluated in three sites and (6) fourteen ESWYT combined datasets evaluated in fourteen sites.

For 99 datasets (except the large S1 irrigated-BP combined dataset), we performed GWAS using the mixed linear model^59^ in TASSEL version 5, where population structure was used as a fixed effect and the kinship or the degree of relatedness between the lines was used as a random effect. We used the first two principal components^60^ to account for the population structure and the pedigree-relationships among the lines to account for kinship. The mixed linear model was run with the optimum level of compression and the population parameters previously determined^61^ options. In the S1 irrigated-BP combined dataset that had 50,283 lines with good genotyping data and was computationally demanding, we evaluated the Bayesian-information and Linkage-disequilibrium Iteratively Nested Keyway (BLINK) method, that is proven to result in higher statistical power and computing efficiency in large datasets^53^. We implemented the BLINK algorithm in the ‘BLINK-R’ package and accounted for population structure using the first two principal components.

To correct for multiple testing and to identify significant markers, we used the Bonferroni correction for multiple testing with an α level of 0.01 for the seven large S1 datasets and a relaxed α level of 0.20 for all the other datasets. The p-values for each marker were obtained in the different datasets and the CMplot ‘R’ package^62^ was used for generating the Manhattan plots. From the markers that were significant after correcting for multiple testing, we obtained the consistent markers that were significant in more than one dataset and their genetic positions on the POPSEQ map^23^. We then obtained 159 markers that were significant in greater than seven datasets, created a reference map aligned to the RefSeq v.1.0 and visualized it using Phenogram (http://visualization.ritchielab.org/phenograms/plot). Since several significant markers were in high LD with nearby markers, we calculated the pairwise R^2^ (a measure of LD) between the markers using all the 50,283 lines and used two criteria (pairwise R^2^ greater than 0.9 and the p-value for the existence of LD equal to zero) to designate markers into an LD-block. However, it should be noted that several LD-blocks could be in LD with the same QTL. The LD-blocks that overlapped between the irrigated-BP environment and the other environments were visualized using the ‘R’ package, ‘VennDiagram’^63^. We also analyzed the additive effects of all the markers from the mixed linear model and obtained the maximum additive effect of a marker in the LD-block, considering only the individual panels, where the additive effect was the effect within an environment. Finally, the position of each significant marker was compared to previously reported markers for GY, GY components and phenology, whose positions were available in the RefSeq v1.0.

### Genomic profiling of lines for grain yield favorable alleles

For the 801 consistent GY-associated markers identified in this study, we profiled 73,142 wheat breeding lines, comprising 62,581 lines from Stage 1 of yield testing developed during 2013–2019, 961 lines from Stage 2 evaluated in the 2013–2014 cycle and 9,600 lines from other CIMMYT populations that were genotyped. For each marker, we obtained the favorable allele (FA) or the allele that had an increasing effect on GY in the significant datasets, the non-favorable allele or the allele that had a decreasing effect on GY in the significant datasets and the heterozygote. We then calculated the percentage of GY FAs in each line and also identified lines that had a high percentage of FAs (greater than 80%) at the non-missing markers and also high GY (over 8.2 t/ha). To visualize the GY FA composition of lines, we took a subset of the consistently significant markers (68 markers that were significant in more than 10 datasets), plotted a heatmap of the alleles and clustered the lines based on the presence of FAs using the ‘R’ package gplots^64^. Furthermore, to understand the percentage of lines with GY FAs at each of the 801 consistent markers, we plotted the percentage of lines with FAs at each marker in the different homeologous chromosomes using the ‘R’ package ‘ggplot2’^65^.

## Supplementary Information

Supplementary Information 1.

Supplementary Figure 1.

Supplementary Figure 2.

Supplementary Figure 3.

Supplementary Figure 4.

Supplementary Figure 5.

Supplementary Figure 6.

Supplementary Figure 7.

Supplementary Figure 8.

Supplementary Figure 9.

Supplementary Figure 10.

Supplementary Tables.

## Data Availability

The phenotyping data for the lines used in this study are available in Supplementary Table 1. The genomic profiles of 73,142 wheat lines for the 801 consistent grain yield associated markers is available in https://hdl.handle.net/11529/10548546.
