# Supplementary material for: Elucidating the genetics of grain yield and stress-resilience in bread wheat using a large-scale genome-wide association mapping study with 55,568 lines
Source: Sci Rep. 2021 Mar 4;11:5254. doi: 10.1038/s41598-021-84308-4 (PMC7933281; doi:10.1038/s41598-021-84308-4)
Supplement: Supplementary file 1 — Supplementary Information 1. [file 41598_2021_84308_MOESM1_ESM.docx]

**Elucidating the genetics of grain yield and stress-resilience in bread wheat using a large-scale genome-wide association mapping study with 55,568 lines**

Philomin Juliana^1^, Ravi Prakash Singh^1^*, Jesse Poland^2^, Sandesh Shrestha^2^, Julio Huerta-Espino^3^, Velu Govindan^1^, Suchismita Mondal^1^, Leonardo Abdiel Crespo-Herrera^1^, Uttam Kumar^4,5^, Arun Kumar Joshi^4,5^, Thomas Payne^1^, Pradeep Kumar Bhati^4,5^, Vipin Tomar^5,6^, Franjel Consolacion^1^ and Jaime Amador Campos Serna^1^

^1^International Maize And Wheat Improvement Center (CIMMYT), Texcoco, Mexico; ^2^Wheat Genetics Resource Center, Department of Plant Pathology, Kansas State University, Manhattan, KS, USA; ^3^Campo Experimental Valle de Mexico, Instituto Nacional de Investigaciones Forestales, Agricolas y Pecuarias (INIFAP), Chapingo, Mexico; ^4^CIMMYT, NASC complex, New Delhi, India; ^5^Borlaug Institute for South Asia (BISA), New Delhi, India; ^6^Institute of Advanced Research, Gandhinagar, Gujarat, India.

**Corresponding author:** Ravi Prakash Singh, [**r.singh@cgiar.org**](mailto:r.singh@cgiar.org)

### Supplementary Fig. 1 | Markers significantly associated with grain yield in the combined Stage 1 (S1) yield trials evaluated in the irrigated-bed planting (BP) environment, best-linear unbiased estimates of S1 and S2 yield trials evaluated in the irrigated-BP environment during the 2012-2013 (1213) to 2018-2019 (1819) cycles and combined (com.) analysis and Stage 3 (S3) yield trials evaluated in the irrigated-BP, late-heat and severe-drought (sev-drought) environments during the 2014-2015 (1415) to 2017-2018 (1819) cycles and combined (com.) analysis. The chromosomes are shown in the x-axis and the -log_10_ p-values in the y-axis. The threshold lines correspond to the threshold using the Bonferroni correction for multiple testing at an α level of 0.20, except for the S1 irrigated-BP com. dataset where the α level was 0.01.

### Supplementary Fig. 2 | A reference map with 29 grain yield associated markers on chromosomes 1A, 2B, 2D, 3A, 3B, 4A, 4D, 5A, 5D, 6A and 7A, consistent in seven to fourteen datasets and aligned to the reference sequence of bread wheat (RefSeq v.1.0).

### Supplementary Fig. 3| Venn diagrams of linkage disequilibrium blocks that were shared between the irrigated-bed planting environment (blue color) and the other environments (yellow color) including the irrigated-flat planting, moderate-drought, severe-drought, early-heat, late-heat and the target sites. The number of linkage disequilibrium blocks that were only significant in the irrigated-bed planting environment, shared between the irrigated-bed planting environment and the other environment and only in the other environment are shown, along with the percentage of linkage disequilibrium blocks in the other environments that overlapped with the irrigated-bed planting environment.

### Supplementary Fig. 4| Percentage of lines with favorable alleles at the markers significantly associated with grain yield in the group 1 homeologous chromosomes. The x-axis indicates the physical position of the grain yield associated marker in bps in the reference sequence of bread wheat (RefSeq v.1.0).

### Supplementary Fig. 5 | Percentage of lines with favorable alleles at the markers significantly associated with grain yield in the group 2 homeologous chromosomes. The x-axis indicates the physical position of the grain yield associated marker in bps in the reference sequence of bread wheat (RefSeq v.1.0).

### Supplementary Fig. 6 | Percentage of lines with favorable alleles at the markers significantly associated with grain yield in the group 3 homeologous chromosomes. The x-axis indicates the physical position of the grain yield associated marker in bps in the reference sequence of bread wheat (RefSeq v.1.0).

### Supplementary Fig. 7 | Percentage of lines with favorable alleles at the markers significantly associated with grain yield in the group 4 homeologous chromosomes. The x-axis indicates the physical position of the grain yield associated marker in bps in the reference sequence of bread wheat (RefSeq v.1.0).

### Supplementary Fig. 8 | Percentage of lines with favorable alleles at the markers significantly associated with grain yield in the group 5 homeologous chromosomes. The x-axis indicates the physical position of the grain yield associated marker in bps in the reference sequence of bread wheat (RefSeq v.1.0).

### Supplementary Fig. 9 | Percentage of lines with favorable alleles at the markers significantly associated with grain yield in the group 6 homeologous chromosomes. The x-axis indicates the physical position of the grain yield associated marker in bps in the reference sequence of bread wheat (RefSeq v.1.0).

### Supplementary Fig. 10 | Percentage of lines with favorable alleles at the markers significantly associated with grain yield in the group 7 homeologous chromosomes. The x-axis indicates the physical position of the grain yield associated marker in bps in the reference sequence of bread wheat (RefSeq v.1.0).
