# Supplementary figures and images for: Elucidating the genetics of grain yield and stress-resilience in bread wheat using a large-scale genome-wide association mapping study with 55,568 lines
Source: Sci Rep. 2021 Mar 4;11:5254. doi: 10.1038/s41598-021-84308-4 (PMC7933281; doi:10.1038/s41598-021-84308-4)

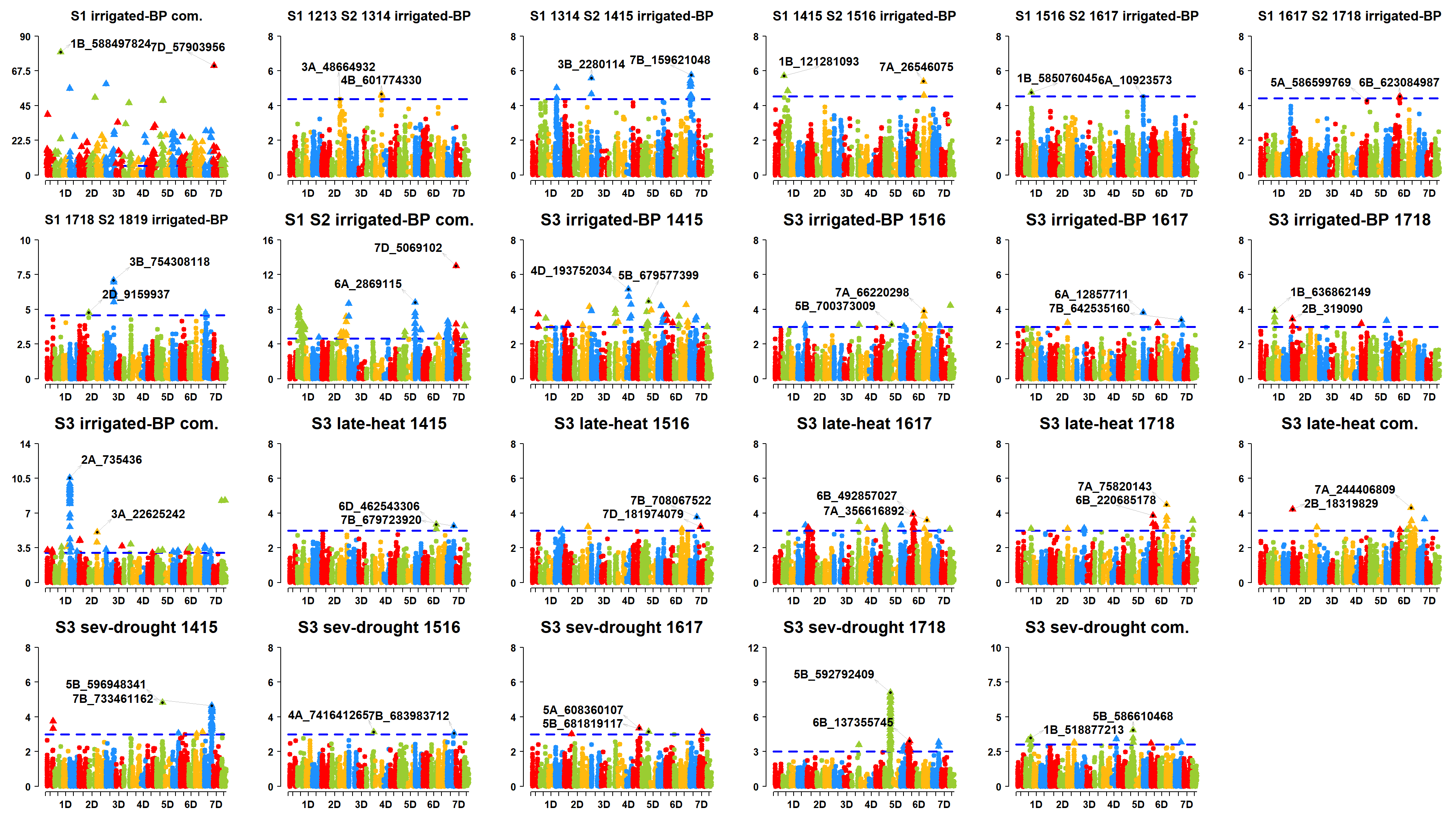

Supplement: Supplementary file 2 — Supplementary Figure 1. [file 41598_2021_84308_MOESM2_ESM.tiff]

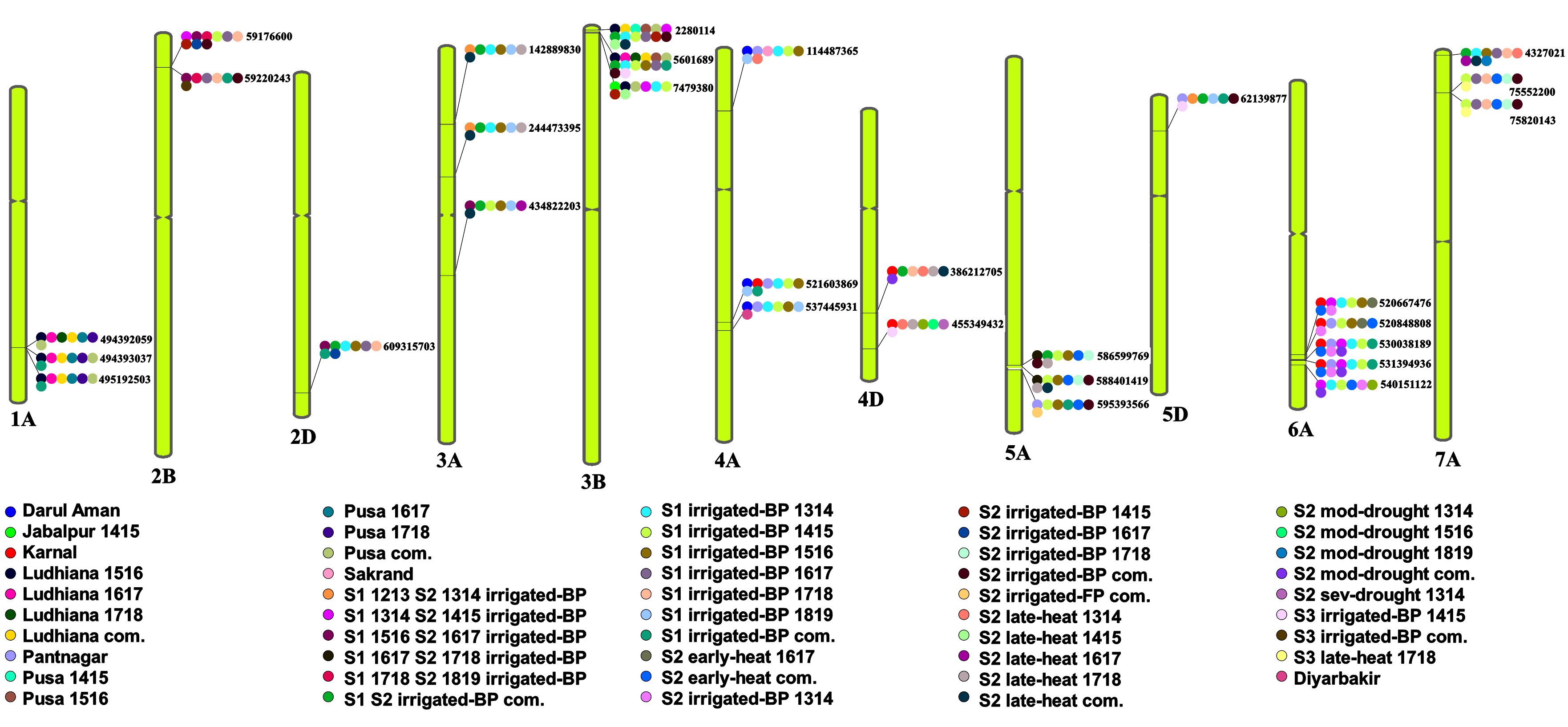

Supplement: Supplementary file 3 — Supplementary Figure 2. [file 41598_2021_84308_MOESM3_ESM.tiff]

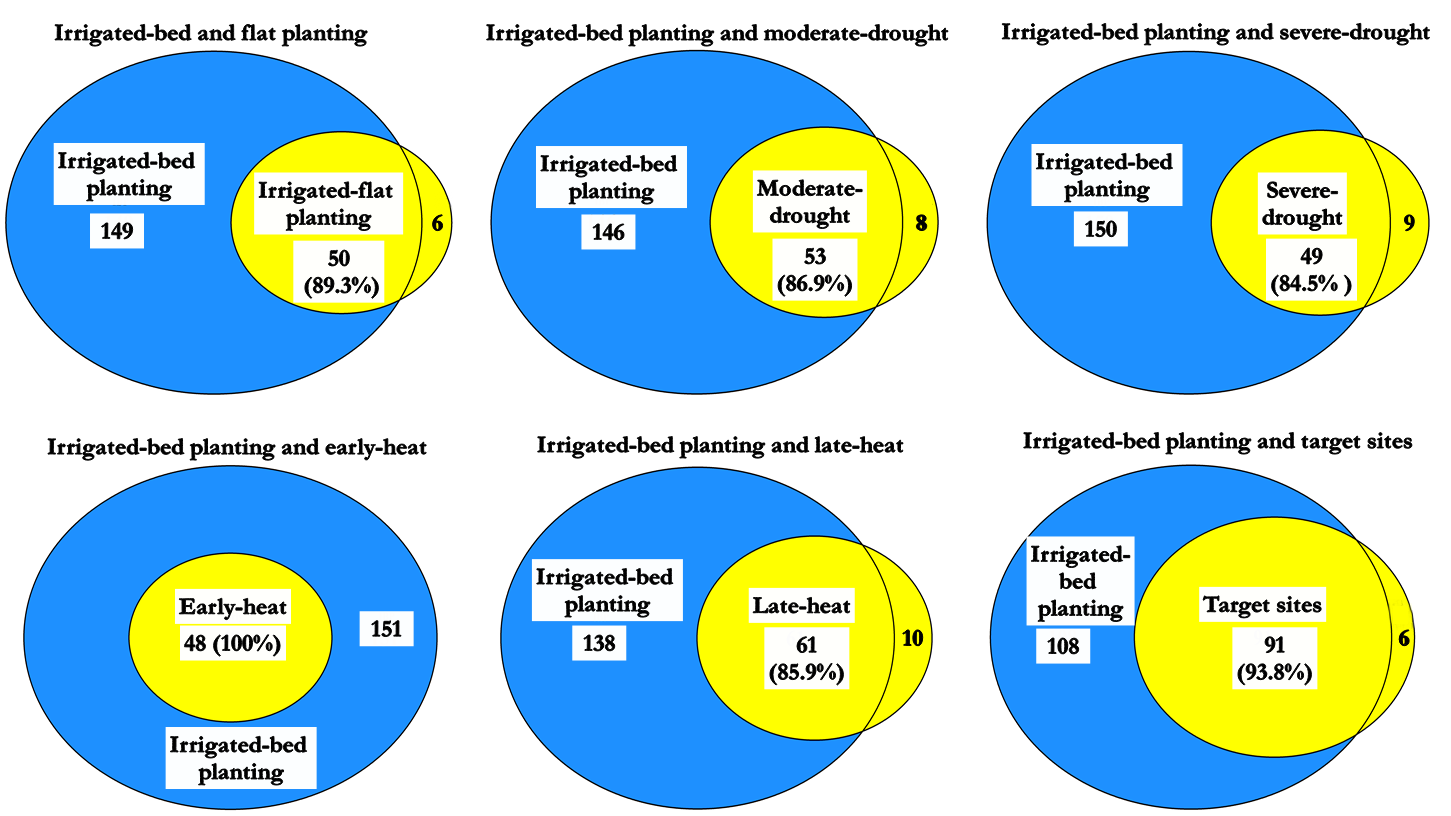

Supplement: Supplementary file 4 — Supplementary Figure 3. [file 41598_2021_84308_MOESM4_ESM.tif]

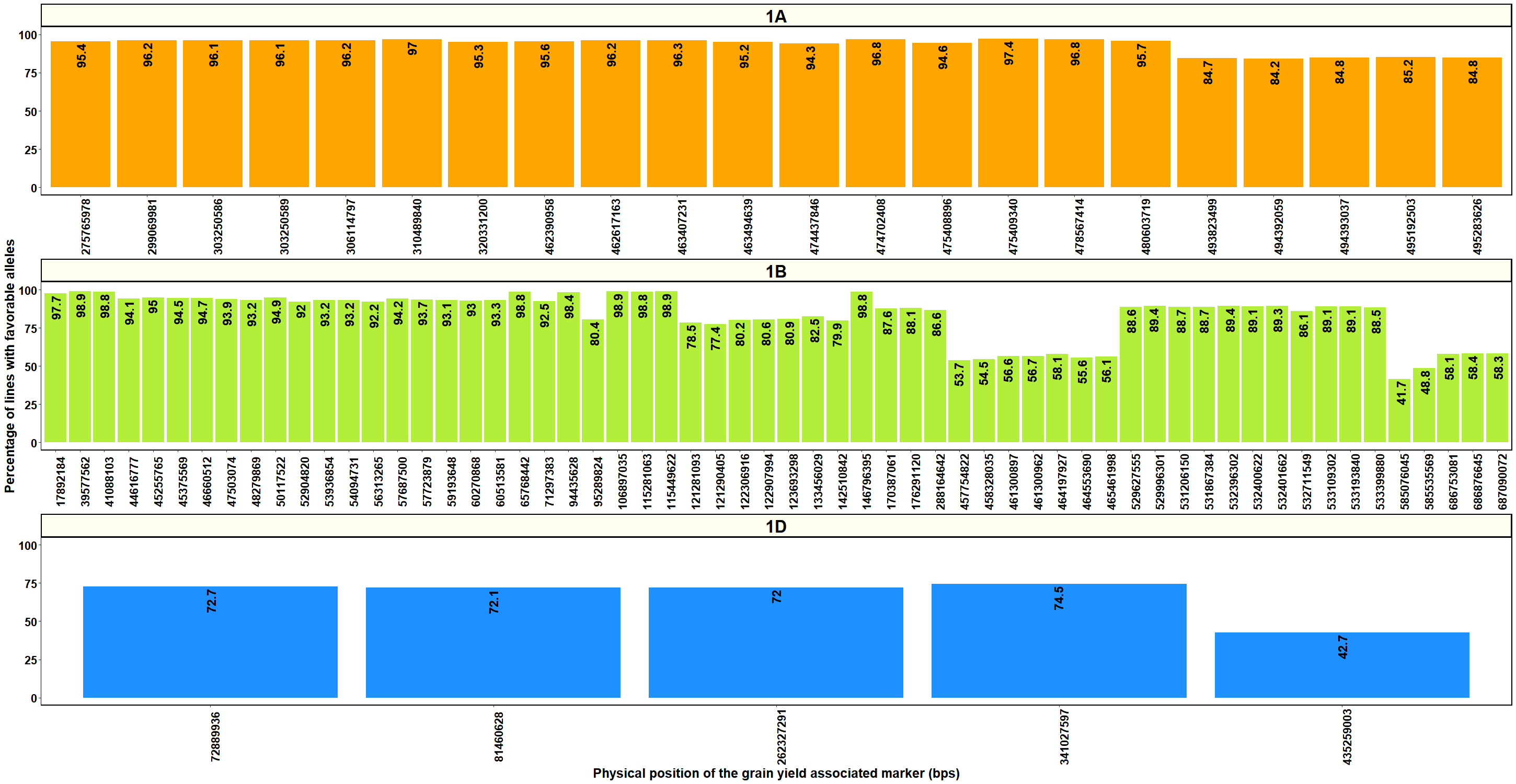

Supplement: Supplementary file 5 — Supplementary Figure 4. [file 41598_2021_84308_MOESM5_ESM.tiff]

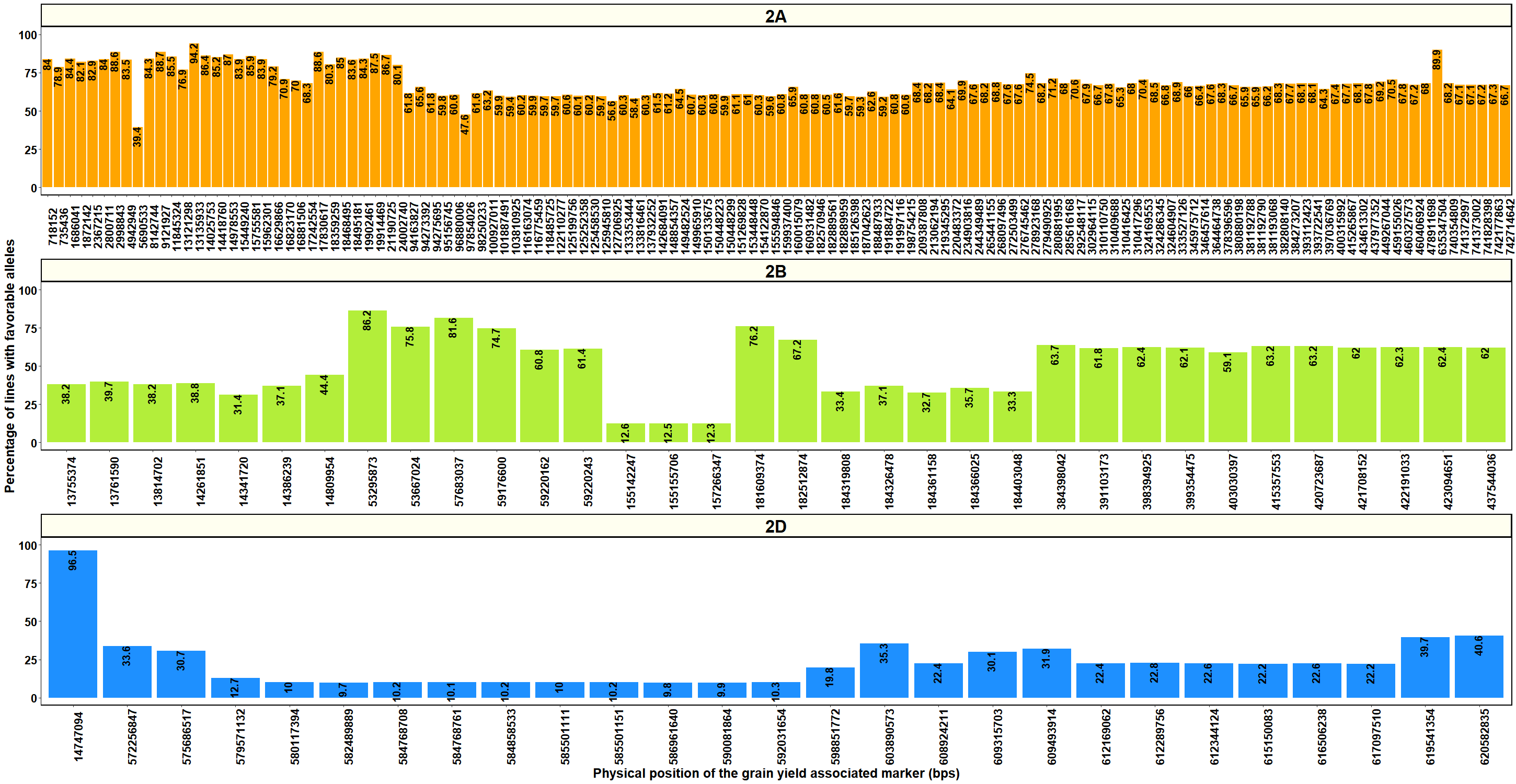

Supplement: Supplementary file 6 — Supplementary Figure 5. [file 41598_2021_84308_MOESM6_ESM.tiff]

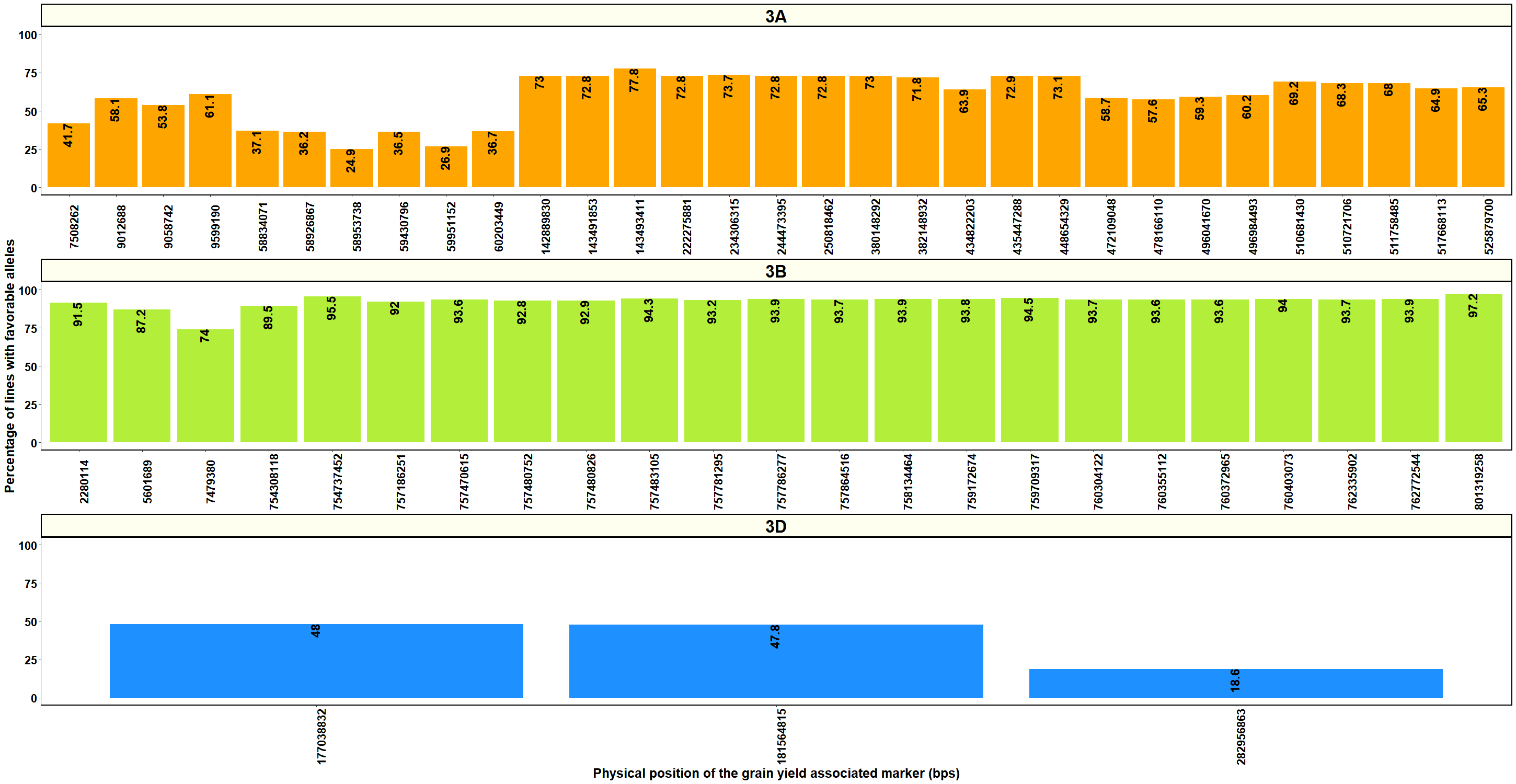

Supplement: Supplementary file 7 — Supplementary Figure 6. [file 41598_2021_84308_MOESM7_ESM.tiff]

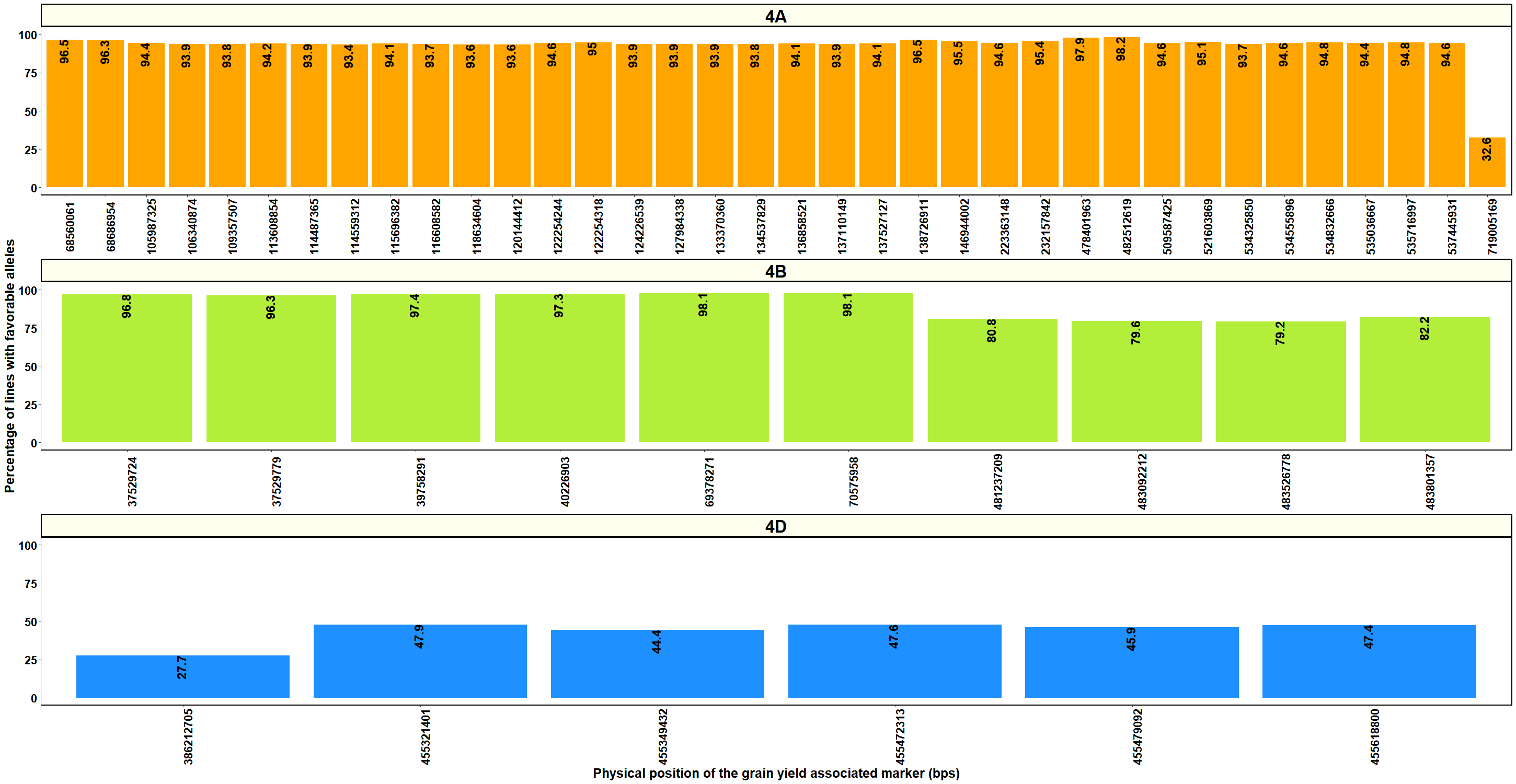

Supplement: Supplementary file 8 — Supplementary Figure 7. [file 41598_2021_84308_MOESM8_ESM.tiff]

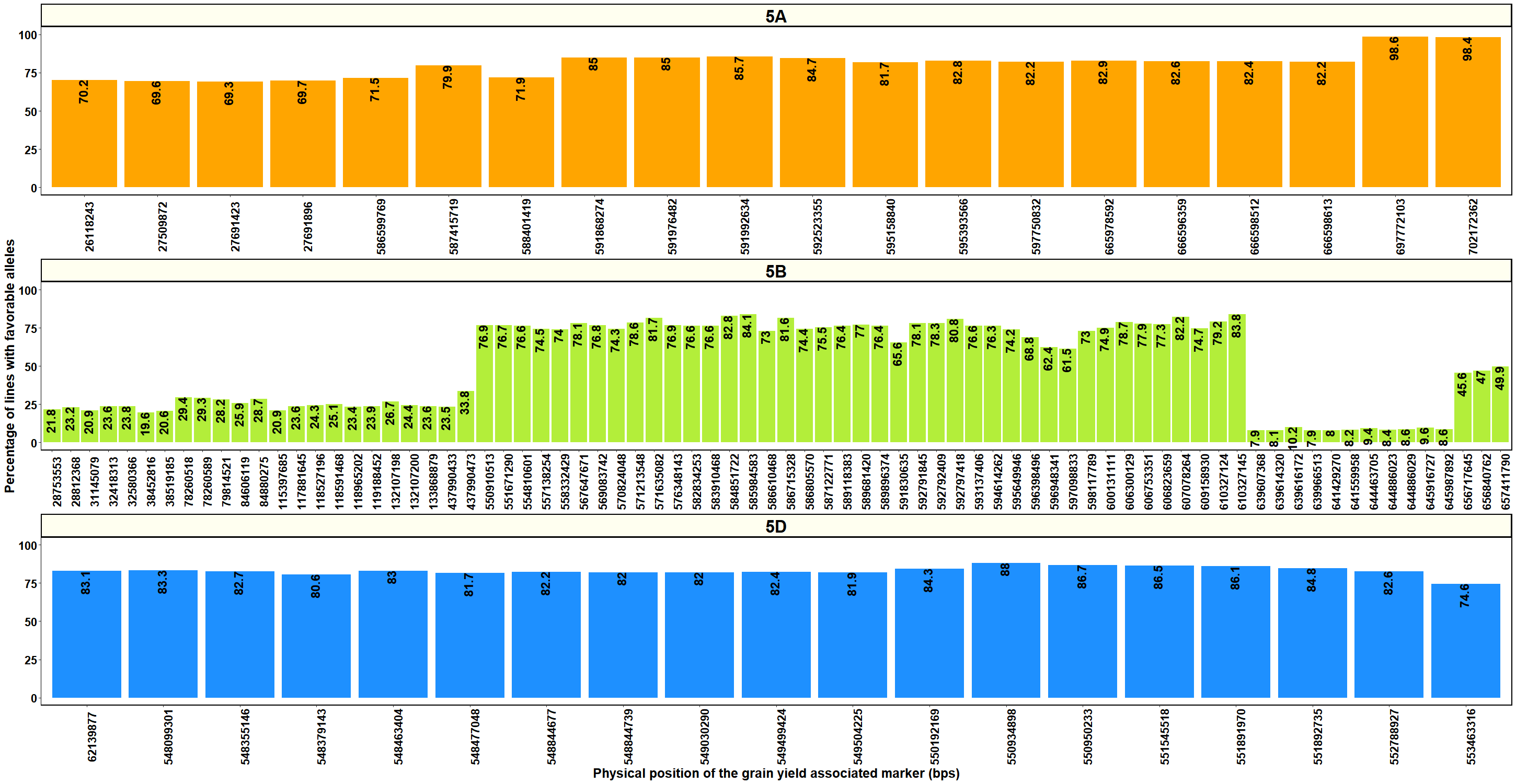

Supplement: Supplementary file 9 — Supplementary Figure 8. [file 41598_2021_84308_MOESM9_ESM.tiff]

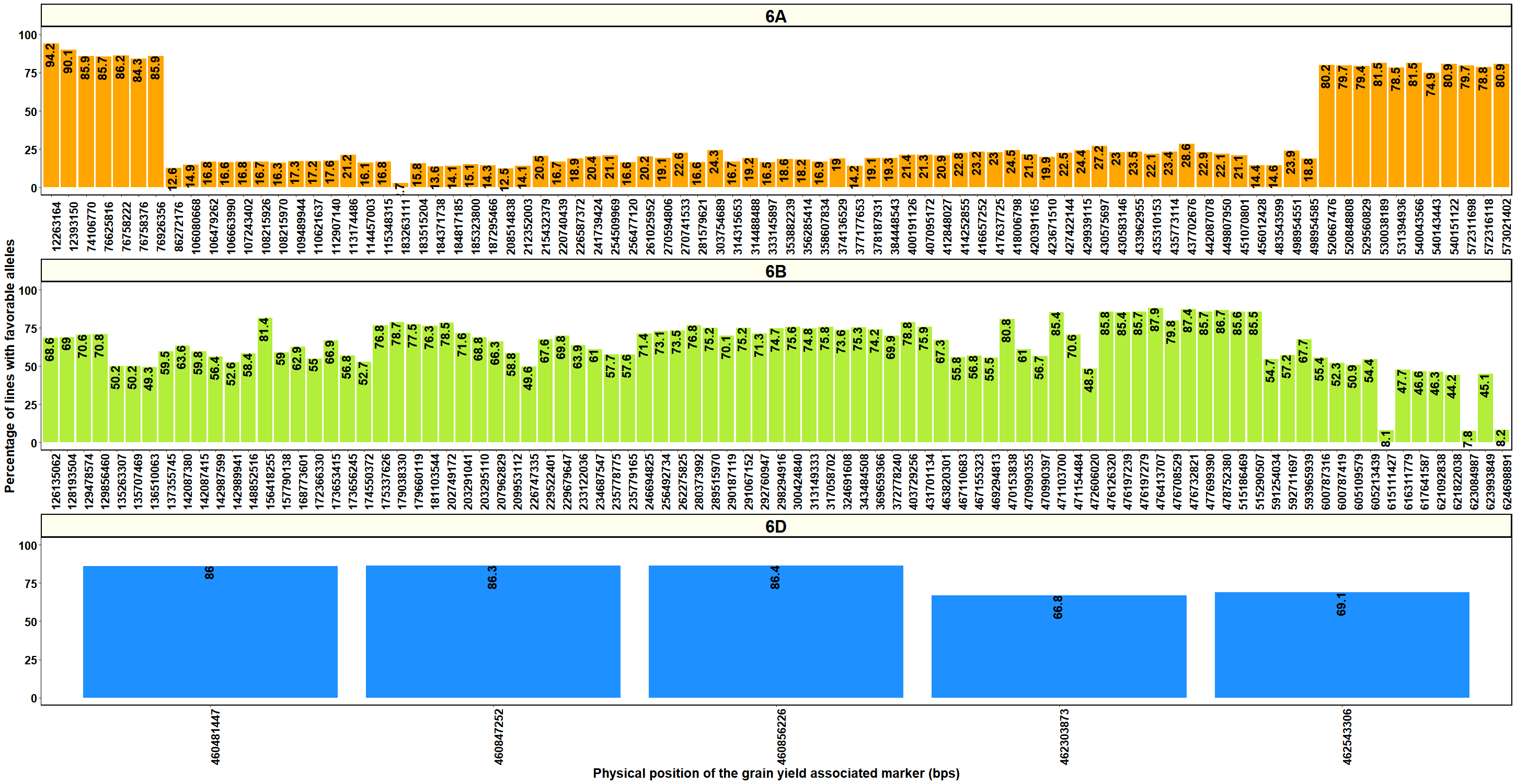

Supplement: Supplementary file 10 — Supplementary Figure 9. [file 41598_2021_84308_MOESM10_ESM.tiff]

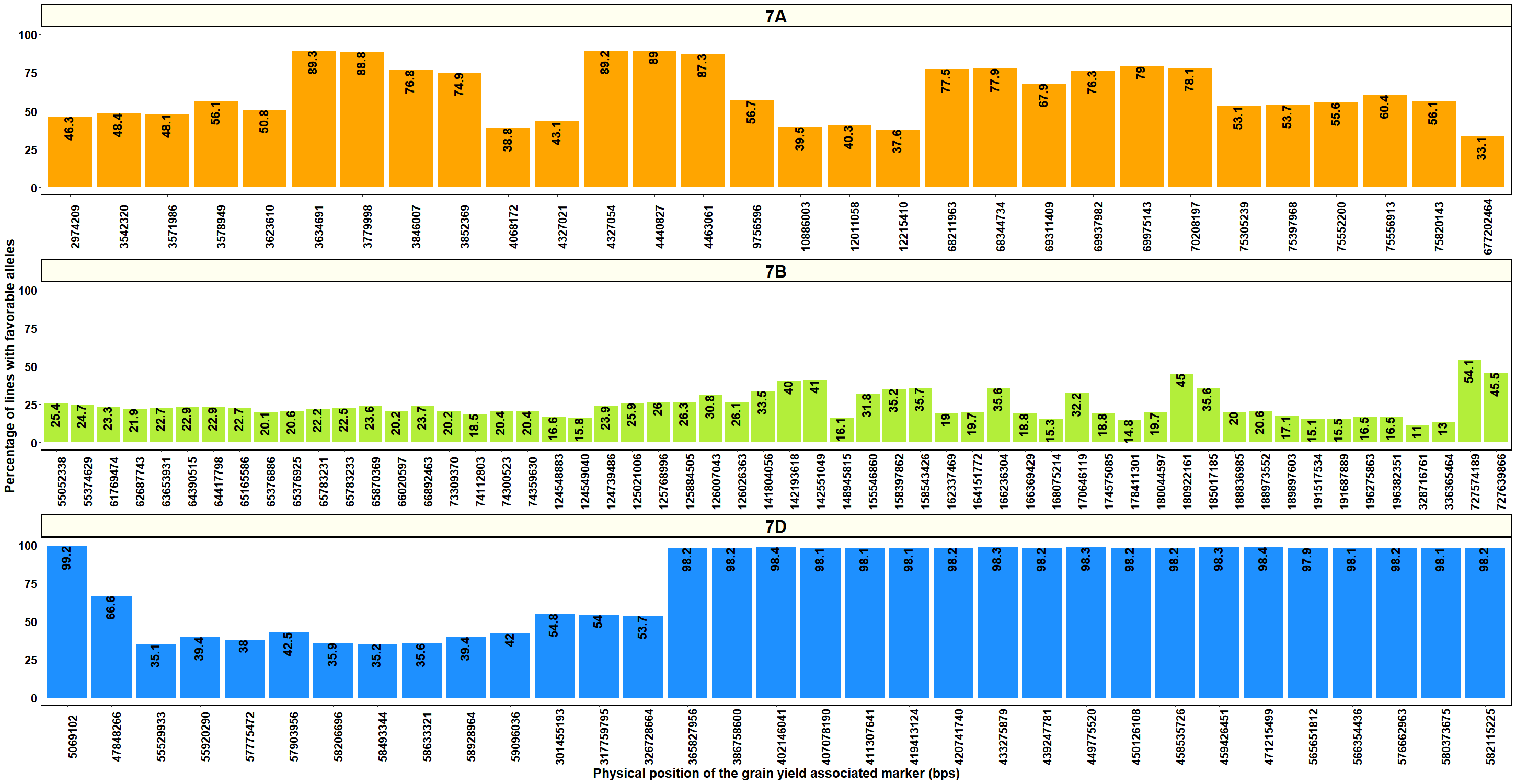

Supplement: Supplementary file 11 — Supplementary Figure 10. [file 41598_2021_84308_MOESM11_ESM.tiff]
